# Molecular characterization and clinical features of diffuse midline glioma in the pediatric precision oncology registry INFORM

**DOI:** 10.1007/s00401-025-02945-9

**Published:** 2025-10-11

**Authors:** Elke Pfaff, Kathrin Schramm, Mirjam Blattner-Johnson, Barbara C. Jones, Sebastian Stark, Gnana Prakash Balasubramanian, Christopher Previti, Robert J. Autry, Petra Fiesel, Felix Sahm, David Reuss, Andreas von Deimling, Cornelis M. van Tilburg, Kristian W. Pajtler, Till Milde, Uta Dirksen, Christof M. Kramm, André O. von Bueren, Caroline Hutter, Bram de Wilde, Jan Molenaar, Nicolas U. Gerber, Olli Lohi, Monica C. Munthe-Kaas, Kleopatra Georgantzi, Bernarda Kazanowska, Michal Zápotocký, Antonis Kattamis, Maria Filippidou, Iris Fried, Stefan M. Pfister, Olaf Witt, David T. W. Jones

**Affiliations:** 1 Hopp Children’s Cancer Center Heidelberg (KiTZ), Heidelberg, Germany; 2 Division of Pediatric Glioma Research, German Cancer Research Center (DKFZ), Heidelberg, Germany; 3 National Center for Tumor Diseases (NCT), NCT Heidelberg, a partnership between DKFZ and Heidelberg University Hospital, Heidelberg, Germany; 4 Department of Pediatric Oncology, Hematology, Immunology and Pulmonology, Heidelberg University Hospital, Heidelberg, Germany; 5 Division of Pediatric Neurooncology, German Cancer Research Center (DKFZ), Heidelberg, Germany; 6 Core Facility Omics IT and Data Management (ODCF), German Cancer Research Center (DKFZ), Heidelberg, Germany; 7 Department of Neuropathology, Institute of Pathology, Heidelberg University Hospital, Heidelberg, Germany; 8 Clinical Cooperation Unit Neuropathology, German Cancer Research Center (DKFZ) and German Cancer Consortium (DKTK), Heidelberg, Germany; 9 Clinical Cooperation Unit Pediatric Oncology, German Cancer Research Center (DKFZ) and German Cancer Consortium (DKTK), Heidelberg, Germany; 10 Department of Pediatrics and Adolescent Medicine, University Hospital Jena, Friedrich Schiller University Jena, Jena, Germany; 11 Pediatrics III, West German Cancer Centre Essen, University Hospital Essen, University of Duisburg-Essen, Essen, Germany; 12 German Cancer Consortium (DKTK) partner site Essen, University Hospital Essen, Essen, Germany; 13 National Center for Tumor Diseases (NCT) partner site Essen, University Hospital Essen, Essen, Germany; 14 Division of Pediatric Hematology and Oncology, University Medical Center Goettingen, Göttingen, Germany; 15 Division of Pediatric Hematology and Oncology, Department of Pediatrics, Gynecology and Obstetrics, University Hospital of Geneva, Geneva, Switzerland; 16 Department of Pediatrics, Gynecology and Obstetrics, Faculty of Medicine, CANSEARCH Research Platform for Pediatric Oncology and Hematology, University of Geneva, Geneva, Switzerland; 17 Department of Pediatrics and Adolescent Medicine, St. Anna Children’s Hospital, Medical University of Vienna, Vienna, Austria; 18 Pediatric Hematology and Oncology, Ghent University Hospital, Ghent, Belgium; 19 Princess Máxima Center for Pediatric Oncology, Utrecht, The Netherlands; 20 Department of Oncology and Children’s Research Center, University Children’s Hospital Zurich, Eleonoren Foundation, Zurich, Switzerland; 21 Faculty of Medicine and Health Technology, Tampere Center for Child, Adolescent and Maternal Health Research, Tampere University, Tampere, Finland; 22 Tays Cancer Center, Tampere University Hospital, Tampere, Finland; 23 Department of Pediatric Oncology and Hematology, Oslo University Hospital, Oslo, Norway; 24 Childhood Cancer Research Unit, Department of Women’s and Children’s Health, Karolinska Institute, Stockholm, Sweden; 25 Division of Pediatric Hematology-Oncology, Astrid Lindgren Children’s Hospital, Karolinska University Hospital, Stockholm, Sweden; 26 Department of Pediatric Hematology/Oncology and BMT, University of Wroclaw, Wrocław, Poland; 27 Center for Pediatric Neuro-Oncology, Motol University Hospital, Prague, Czech Republic; 28 Department of Paediatric Haematology and Oncology, Second Faculty of Medicine, Charles University and Motol University Hospital, Prague, Czech Republic; 29 Division of Pediatric Hematology-Oncology, First Department of Pediatrics, “Aghia Sophia” Children’s Hospital, National and Kapodistrian University of Athens, Athens, Greece; 30 “Aghia Sophia” Children’s Hospital ERN-PeadCan Center, Athens, Greece; 31 Pediatric Hemato-Oncology Unit, Wilf Children’s Hospital, Shaare Zedek Medical Center, Jerusalem, Israel; 32 Faculty of Medicine, Hebrew University of Jerusalem, Jerusalem, Israel

**Keywords:** Pediatric diffuse midline glioma, Molecular characterization, Targeted therapy, Predictive and prognostic markers

## Abstract

Diffuse midline glioma (DMG; a subtype of pediatric high-grade glioma) is a fatal disease in children, due to the localization in critical structures of the central nervous system, its invasive nature, and limited treatment options. Molecularly, DMG with loss of histone 3 K27 trimethylation (mostly through the typical K27M-mutation in histone 3) have been relatively well characterized, however, no unambiguous Achilles’ heel for targeted therapeutic approaches could be identified to date. This study integrates detailed molecular characteristics of pediatric DMGs with clinical data in a large, international cohort in order to contribute to a better understanding necessary for further development of therapeutic approaches. A total of 162 DMG tumors were analyzed within the INFORM registry from 01/2015 to 11/2023 using comprehensive molecular profiling (including exome, whole-genome and RNA next-generation sequencing approaches, complemented with DNA methylation analysis). Molecular results were correlated with clinical data of the respective patients including the treatment regimen applied and patients’ outcomes. This well-defined cohort of histone 3 K27-altered DMG according to the current WHO classification showed typical molecular alterations for this entity, with differences in frequencies in specific subgroups. The presence of *TP53* mutation and the absence of MAPK pathway alteration in the tumors were associated with worse outcomes. In a substantial proportion of patients, genetic alterations serving as targets for potential therapeutic approaches could be identified. This large, international, prospective DMG cohort combines comprehensive molecular characterization of the tumors with registry-level clinical data, thereby contributing to a better understanding of the underlying tumor biology, potential prognostic and predictive markers and the potential impact of targeted therapies.

**Supplementary Information:**

The online version contains supplementary material available at 10.1007/s00401-025-02945-9.

## Introduction

Diffuse midline glioma (DMG) with histone 3 K27-alteration belongs to the group of pediatric-type diffuse high-grade gliomas (pedHGG) according to the 5th Edition WHO classification of central nervous system tumors, 2021 [31]. The most common localization of DMG in children is the brainstem/pons—corresponding with the clinico-radiological diagnosis of diffuse intrinsic pontine glioma (DIPG). Less frequently, DMG arise in the thalamic region (uni- or bilaterally), the spinal cord, or rarely in other midline structures like the pineal region, hypothalamus, or cerebellum/cerebellar peduncles [42]. Prognosis for DMG patients in general is very poor, with a 2-year-survival rate of less than 10% [33]. Several specific features of DMG contribute to the unfavorable clinical course: (i) the involvement of vital brain regions, like the pons, (ii) limited surgical options due to the infiltrative growth pattern and the tumor localization in critical structures, and (iii) scarce further treatment options with conventional treatment including radio- and chemotherapy [32].

Molecularly, DMG are characterized by loss of histone 3 K27 trimethylation (H3 K27me3) [4, 12, 30], in most tumors through an *H3F3A* K27M mutation and less frequently a similar substitution in another histone H3 isoform or overexpression of *EZHIP*. A related tumor class is described as having either an H3 mutation or *EZHIP* overexpression together with frequent activating alterations of *EGFR* and typical (bi-)thalamic location [36]. These different alterations define four subtypes of H3 K27-altered DMG [31] (H3.1 K27-mutant, H3.3 K27-mutant, H3-wildtype with *EZHIP* overexpression, *EGFR*-altered). Pathogenetically, loss of H3 K27me3 is assumed to be caused by the inhibitory interaction between the characteristic H3 K27M mutation and EZH2 (methyltransferase catalytic subunit of PRC2). In H3-wildtype tumors, inhibition of the PRC2 complex is induced by the EZHIP protein that naturally mimics the amino acid structure of a histone tail with the K27M mutation [9, 27]. Through large-scale biological studies conducted in the past years, DMG and DIPG are nowadays relatively well characterized molecularly [10, 11, 14, 33]. Besides the characteristic H3 mutations, alterations in the p53 pathway, including *TP53* and *PPM1D*, are found especially in the H3.3-mutant and *EGFR*-altered subtypes. Conversely, PI3K and MAPK pathways are more frequently affected in the subtypes harboring H3.1/H3.2 mutation. Further alterations described in DMG include mutation or amplification of *PDGFRA*, *FGFR1,* or *ACVR1,* as well as *BRAF* V600E mutation (more commonly found in pediatric low-grade glioma) in the H3.3-mutant subtype [5, 19, 46, 52, 53].

According to the WHO classification, DMG H3 K27-altered is a molecular-pathologically defined entity, however, this diagnosis includes a heterogeneous group of tumors regarding localization, mechanism of loss of K27me3 and concomitant molecular alterations. This contributes to the fact that DMG in children are among the most difficult-to-treat pediatric brain tumors.

We compiled the molecular data from 162 DMG patients investigated within the INFORM registry (Individualised Therapy For Relapsed Malignancies In Childhood) [17–19] from 01/2015 to 11/2023 and correlated the molecular characteristics with clinical and outcome data. The aim of the study is to gain further knowledge on the molecular landscape and the clinical behavior of the different subtypes of DMG with H3 K27-alteration which could form a basis for the further development of therapeutic approaches.

## Materials and methods

Pediatric and adolescent patients with relapsed, progressive, or high-risk malignancies can be enrolled in the international, multi-center, prospective, non-interventional INFORM registry (https://www.kitz-heidelberg.de/en/clinical-studies/inform), which provides comprehensive molecular characterization of the respective tumors. The INFORM registry is listed in the German Clinical Trials Register (Study ID: DRKS00007623) and the protocol was reviewed and approved by the Ethics Committee of the Heidelberg University Hospital, as well as respective ethic committees in participating countries. Patients or the legal representatives consented to registration in the INFORM registry, to the conduction of molecular analysis of the tumor and constitutional DNA and to the scientific evaluation of molecular and clinical data obtained through the participation in the INFORM registry. The study was conducted in accordance with Good Clinical Practice guidelines and the Declaration of Helsinki. The study presented here includes data which was generated and provided in the scope of the INFORM registry [26, 48, 51].

For inclusion in the current study, patients registered in the INFORM registry with the diagnosis of HGG/DIPG were evaluated. For a subset of tumors analysis could not be performed, because INFORM inclusion criteria were not fulfilled, tumor material was not sent after registration or material was not suitable for analysis. Furthermore, cases with one of the following characteristics were excluded from the current cohort: (i) repetitive analysis of samples from the same patient (note: none of the DMG tumors was re-analyzed), (ii) early pilot patients without available follow-up data, (iii) localization of the tumor clearly in non-midline structures (specification see below), (iv) histological diagnosis of different HGG subgroup, (v) tumor cell content in sample too low for analysis/evaluation, (vi) detection of IDH1-mutation, (vii) detection of histone 3 G34-mutation, (viii) high classification score (> 0.9) based on DNA methylation analysis for different HGG subgroup (even with localization in midline structures), (ix) allocation to different HGG subgroup based on DNA methylation profile and clustering in t-SNE with respective reference cohorts (without unequivocal classification score), (x) no clear subgroup affiliation possible. The latter criterion refers to cases that could not be clearly allocated to any molecular subgroup based on methylation analysis, taking into account the classification score using version v12.8 of the “Heidelberg classifier” as well as t-SNE clustering with a large reference cohort. Tumors harboring a K27M mutation in one of the histone isoforms and/or with a clear classification score for the DMG K27-altered subgroup (including DMG with *EGFR* alteration) were included even if the localization of the tumor was unclear or not unequivocally restricted to midline structures.

The INFORM pipeline, including whole-exome sequencing (WES), low-coverage whole-genome sequencing (lc-WGS), RNA sequencing and DNA methylation analysis on tumor and constitutional material (WES and lc-WGS) has been described previously [39, 48, 51]. Data will be made available via the German Human Genome-Phenome Archive (GHGA, https://www.ghga.de/). Data were processed bioinformatically according to protocols established for the INFORM pipeline [39, 48, 51]. For evaluation of DNAmethylation-based classification and assignment of tumors to the respective molecular brain tumor (sub-)groups, version v12.8 of the “Heidelberg classifier” was used as previously described [7] (https://app.epignostix.com). For clear subgroup assignment, the threshold score was set at > 0.9. Statistical comparison of different subgroups regarding specific alteration frequencies was conducted using R version 4.4.2 [47] applying Fisher’s exact test. For gene expression analysis based on RNA sequencing, the R2 Genomics Analysis and Visualization Platform (https://hgserver1.amc.nl/cgi-bin/r2) was used. *EZHIP* expression with at least two-fold increase compared to the other tumors within the group of DMG and pedHGG with available expression data (*n* = 190) was defined as *EZHIP* overexpression. Genes differentially expressed between different subgroups were identified by t-test. Gene set enrichment analysis was performed using the GSEA 4.4.0 software (https://www.gsea-msigdb.org/gsea/index.jsp) [44] and applying the ‘oncogenic signature gene sets’ of the Human MSigDB Collections.

The genomic signature for alternative lengthening of telomeres (ALT) was determined using the previously outlined software tool TelomereHunter [18, 41] based on low-coverage whole genome profiling (lcWGS).

Information on the past medical history, applied treatment, as well as on follow-up of the respective patients was collected by the treating pediatric oncology centers and made available through an electronic Case Report Form (eCRF). Survival analyses were performed using R version 4.4.2 [47]. For the determination of overall survival (OS) and progression-free survival (PFS) stratified for different subgroups within the cohort, the Kaplan–Meier method was applied. Differences between the Kaplan–Meier curves were identified by log-rank test (*p*-value). OS of the current episode was defined as the time between diagnosis of the episode (primary diagnosis, refractory disease, relapse) leading to enrollment in the INFORM registry and the last follow-up or death. PFS of the current episode was defined as time between diagnosis of the episode (primary diagnosis, refractory disease, relapse) leading to enrollment in the INFORM registry and further/first progression or death (if death occurred before the first response assessment scheduled 3 months after INFORM analysis). Radiologic response evaluation was performed locally at the treating center. OS of primary diagnosis was defined as the time between primary diagnosis and last follow-up or death. For univariate and multivariate analysis, a Cox regression model was applied using R version 4.4.2 [47] reporting results as hazard ratios (HR), respective 95% confidence intervals (CI) and *p*-value obtained by log-rank test.

## Results

Since the initiation of the INFORM registry in 01/2015 until the data cut-off for the current study 11/2023, a total of about 3000 patients spanning all entities have been enrolled. Based on the above-mentioned selection criteria, 162 patients belonging to the DMG K27-altered subgroup were included in the current study (Supplementary Fig. 1). The patients were enrolled by 33 German pediatric oncology centers, as well as by centers in Austria, Belgium, the Netherlands, Switzerland, Sweden, Norway, Finland, Poland, the Czech Republic, Greece and Israel.

Median patient age at the episode of the disease leading to enrollment in INFORM was 8 years, with 12.3% of patients being 4 years of age or less. Pons/brainstem was the most frequent tumor localization (60.5% of patients), followed by the thalamic region (11.7%). Around three-quarters of patients (75.9%) had localized disease at the current episode. Most patients (*n* = 126; 77.8%) were enrolled at primary diagnosis, whereas INFORM analysis was performed due to refractory disease or progression under first-line treatment in 15 patients. For the 10 patients registered at first relapse (after completion of first-line treatment) the median latency from primary diagnosis to relapse was 10.5 months (range 0.9–47.9 months), whereas eight patients suffered from second or multiple relapses with a median latency of the current episode from primary diagnosis of 29.9 months (range 7.5–91.4 months). In 60.5% of patients, only a biopsy was performed at the current episode to obtain tumor material, with the remainder undergoing some form of resection. Details on clinical characteristics for the whole cohort as well as separated for primary and relapse/progressive disease are provided in Table 1.

**Table 1:** Details on clinical features of the study cohort

Clinical characteristics	Total (*n* = 162)	Primary (*n* = 126)	Relapse/progression (*n* = 33)
Missing data regarding episode	3 (1.9%)		
**Sex, no. (%)**			
Female	77 (47.5%)	61 (48.4%)	15 (45.4%)
Male	85 (52.5%)	65 (51.6%)	18 (54.5%)
**Age (current episode), no. (%)**			
≤ 4 yr	20 (12.3%)	18 (14.3%)	2 (6.1%)
> 4 yr	139 (85.8%)	108 (85.7%)	31 (93.9%)
Age, median yrs (range)	8 (2–22)	7 (2–22)	10 (3–18)
Missing data	3 (1.9%)		
**Age (primary diagnosis), no. (%)**			
≤ 4 yr	22 (13.6%)		4 (12.1%)
> 4 yr	137 (84.6%)		29 (87.9%)
Age, median yrs (range)	7.5 (1–22)		9 (1–17)
**Localization, no. (%)**			
Thalamus	19 (11.7%)	16 (12.7%)	3 (9.1%)
Basal ganglia	2 (1.2%)	1 (0.8%)	1 (3.0%)
Pons/brainstem	98 (60.5%)	82 (65.1%)	14 (42.4%)
Spinal	9 (5.6%)	4 (3.2%)	5 (15.2%)
Missing data/unclear	34 (21.0%)	23 (18.3%)	10 (30.3%)
**Performance status (Karnofsky/Lansky), no. (%)**			
< 50%	1 (0.6%)	1 (0.8%)	0
50–60%	19 (11.7%)	13 (10.3%)	6 (18.2%)
70–80%	75 (46.3%)	58 (46.0%)	17 (51.5%)
90–100%	52 (32.1%)	44 (34.9%)	8 (24.2%)
Missing data	15 (9.3%)	10 (7.9%)	2 (6.1%)
**Number of relapse, no. (%)**			
Primary diagnosis	126 (77.8%)		
Refractory to therapy/progression under first-line therapy	15 (9.3%)		
1. Relapse	10 (6.2%)		
2. Relapse	5 (3.1%)		
≥ 3. Relapse	3 (1.9%)		
Missing data	3 (1.9%)		
Median latency after previous episode for relapses (range) [months]	9.0 (3.6–84.5)		
**Metastatic status [**25, 37**], no. (%)**			
M0	123 (75.9%)	104 (82.5%)	19 (57.6%)
M1	1 (0.6%)	0	1 (3.0%)
M2/M3/M+	13 (8.0%)	4 (3.2%)	9 (27.3%)
Missing data	25 (15.4%)	18 (14.3%)	4 (12.1%)
**Level of resection, no. (%)**			
Biopsy (R4)	98 (60.5%)	84 (66.7%)	14 (42.4%)
Partial resection (R3)	35 (21.6%)	24 (19.0%)	11 (33.3%)
Rim near total resection (R2)	12 (7.4%)	9 (7.1%)	3 (9.1%)
Gross total resection (R1)	4 (2.5%)	1 (0.8%)	3 (9.1%)
R+	1 (0.6%)	1 (0.8%)	0
Missing data	12 (7.4%)	7 (5.6%)	2 (6.1%)

yr = year; metastatic status (based on references [25, 37]) with M0 = no evidence of tumor dissemination; M1 = microscopic presence of tumor cells in CSF; M2 = gross tumor dissemination in subarachnoid space or ventricles; M3 = gross nodula seeding in spinal subarachnoid space; M+ = metastatic disease but extend of metastatic spread unknown; R+ = residual tumor but extent of resection unknown

### DNA methylation subgrouping of DMG

Genome-wide DNA methylation analysis was performed on 160 tumors and it was not possible for two tumors. Based on methylation profiling using the current version v12.8 of the “Heidelberg classifier” (https://app.epignostix.com), 155 tumors (96.9%) were allocated to the group “DMG H3 K27-altered” (DMG_K27) with 134 tumors having a methylation score > 0.9 for the DMG_K27 group. A total of 149 tumors of the DMG_K27 subgroup harbored the characteristic mutation at position 27 of one of the histone 3 isoforms (histone 3.3 in 129 tumors and histone 3.1 in 20 tumors), leading to lysine to methionine substitution (K27M). Of the six H3 K27-wildtype tumors in this subgroup, overexpression of *EZHIP* was observed in three tumors, one had an *EZH2* single-nucleotide variant and two tumors featured none of these alterations.

Five tumors were classified as “DMG with *EGFR* alteration” (DMG_EGFR), of which four harbored an *EGFR* alteration (single nucleotide variant (SNV), small insertion/deletion (InDel) or amplification), with three of these displaying additional *EZHIP* overexpression and one H3.1 K27M-mutation, respectively. No *EGFR* alteration, but H3.3 K27M mutation was found in the fifth tumor of the DMG_EGFR subgroup.

**Fig. 1:**
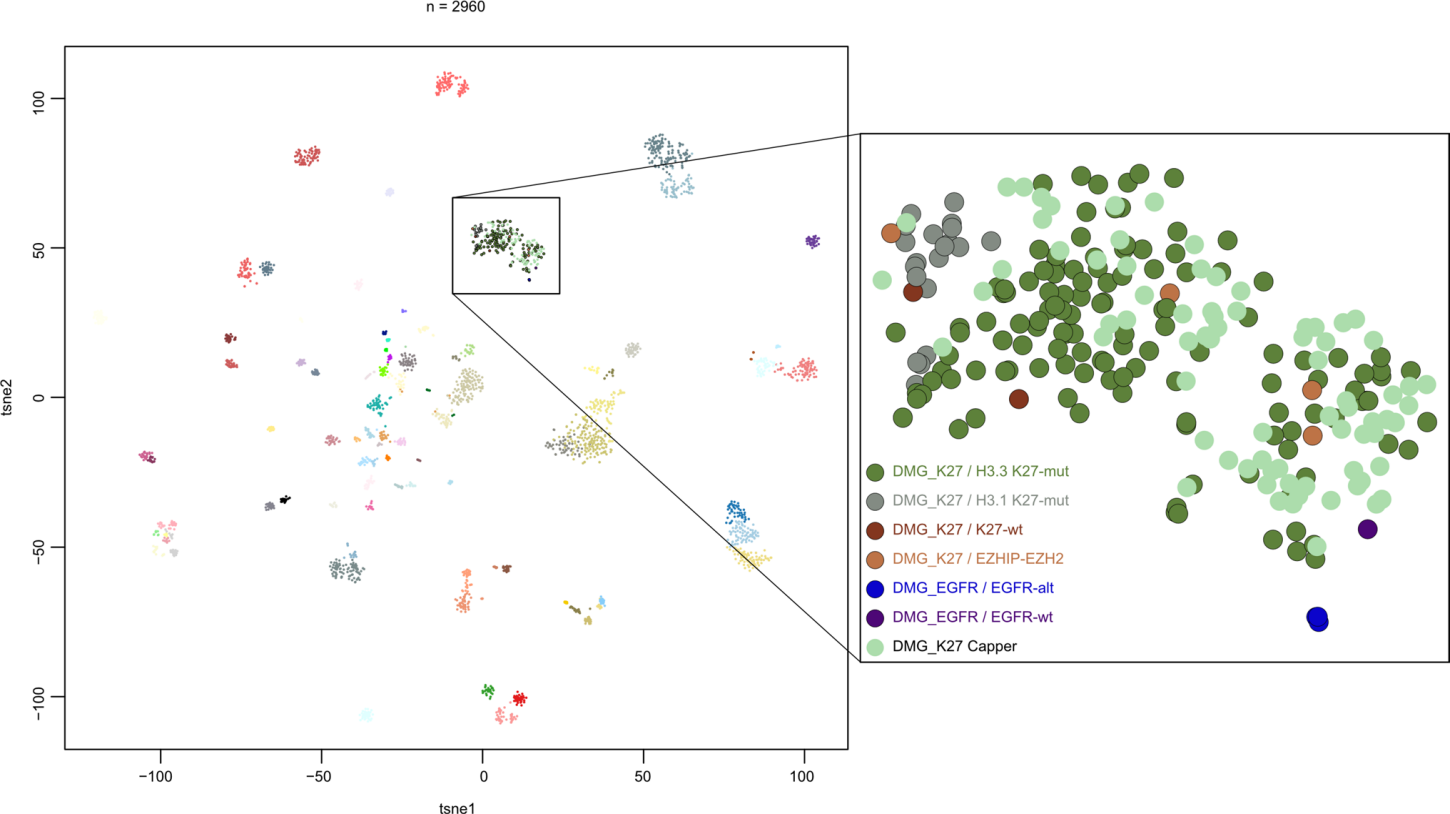
Clustering based on whole-genome DNA methylation analysis visualized by *t*-stochastic neighbor embedding (*t*-SNE) combined with reference cohort from Capper et al. [7]. DMG_K27 Capper = methylation class diffuse midline glioma with Histone 3 K27-alteration included in cohort from Capper; DMG_K27/K27-wt = methylation class diffuse midline glioma Histone 3-altered, without K27M-mutation; DMG_K27/H3.3 K27-mut = methylation class diffuse midline glioma with Histone 3.3 K27M-mutation; DMG_K27/H3.1 K27-mut = methylation class diffuse midline glioma with Histone 3.1 K27M-mutation; DMG_K27/EZHIP-EZH2 = methylation class diffuse midline glioma Histone 3-altered, with *EZHIP* overexpression or EZH2 alteration; DMG_EGFR/EGFR-alt = methylation class diffuse midline glioma *EGFR*-altered, with *EGFR* alteration; DMG_EGFR/EGFR-with = methylation class diffuse midline glioma *EGFR*-altered, without *EGFR* alteration

Visualization by t-stochastic neighbor embedding (t-SNE) was used for comparison of the current cohort with a large CNS tumor reference cohort published by Capper et al. [7] (Fig. 1). All tumors from the current INFORM cohort formed a clear cluster with the DMG K27-altered group of the Capper reference cohort, confirming the molecular subgroup allocation. Within the DMG_K27 cluster on t-SNE, tumors with histone H3.1 K27M-mutation fell together at the edge of the larger cluster. Further, tumors of the DMG_EGFR subgroup with *EGFR* alteration formed a small subcluster.

### Pathways altered in DMG

**Fig. 2:**
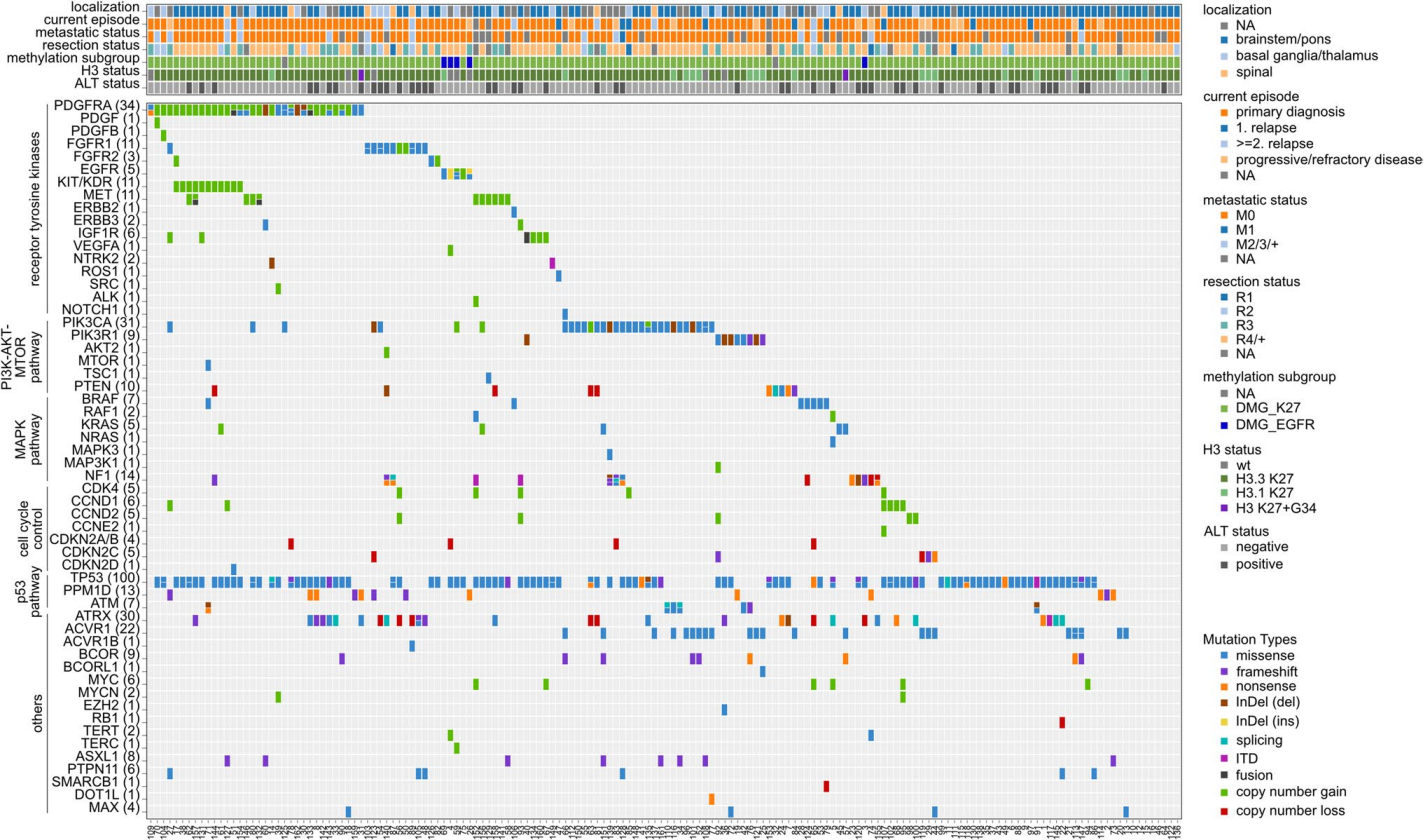
Clinical characteristics and molecular alterations of 162 diffuse midline glioma cases. H3 = Histone 3; ALT = alternative lengthening of telomeres; NA = not available; M0 = no evidence of tumor dissemination; M1 = microscopic presence of tumor cells in CSF; M2 = gross tumor dissemination in subarachnoid space or ventricles; M3 = gross nodula seeding in spinal subarachnoid space; M+ = metastatic disease but extend of metastatic spread unknown; R4 = biopsy; R3 = partial resection; R2 = rim near total resection; R1 = gross total resection; R+ = residual tumor but extent of resection unknown; DMG_K27 = methylation class diffuse midline glioma Histone 3 K27-altered; DMG_EGFR = methylation class diffuse midline glioma *EGFR*-altered; H3.3 K27 = Histone 3.3 K27M-mutation positive; H3.1 K27 = Histone 3.1 K27M-mutation positive; H3 K27 + G34 = H3.3 K27 = Histone 3.3 K27M-mutation and G34-mutation positive; InDel(del) = small deletion; InDel(ins) = small insertion; ITD = internal tandem duplication

The p53 pathway, which is characteristically altered in pedHGG and DMG [33], was also frequently affected in our cohort, with 100 tumors (61.7%; 99 tumors with SNV or InDel, one tumor with intragenic rearrangement) harboring *TP53* alterations, 7 tumors *ATM* mutations and 13 tumors featuring alterations of *PPM1D* (Fig. 2). Alterations in genes coding for receptor tyrosine kinases (RTKs) were identified in *PDGFRA* (*n* = 34), followed by *FGFR1* (*n* = 11), *MET* (*n* = 11) and *EGFR* (*n* = 5). Notably, co-amplification of the nearby located genes *KDR* and *KIT* together with *PDGFRA* amplification—which is a well-known finding in HGG and other tumor entities [8, 16, 35]—was present in eleven tumors. The PI3K-AKT-MTOR pathway was affected most frequently by *PIK3CA* alterations (*n* = 31) as well as *PIK3R1* (*n* = 9) and *PTEN* (*n* = 10). Besides being the pathognomonically altered pathway in pediatric low-grade glioma, alterations in the MAPK pathway (downstream of RTKs) are also described in pedHGG [33]. In the cohort presented here, we identified *BRAF* V600E mutations in five tumors as well as alterations in e.g. *NF1* (*n* = 14) and *KRAS* (*n* = 5). Moreover, 22 tumors (13.6%) harbored alterations in genes playing a role in cell cycle control, e.g. *CDKN2A/B* (*n* = 4), *CDKN2C/CDKN2D* (*n* = 6), *CDK4* (*n* = 5) and *CCND1/CCND2* (*n* = 11) (alterations not mutually exclusive). Further DMG-typical findings included alterations in *ATRX* (*n* = 30) and *ACVR1* (*n* = 22). Whereas, less common in DMG but occasionally observed in our cohort were, for example, *MYC/MYCN* (*n* = 8) and *BCOR* (*n* = 9) alterations. Comparing tumors analyzed at primary diagnosis versus relapsed/progressive tumors, most alterations occurred at a similar frequency during the disease course. *PDGFRA* was more often affected in the tumors at primary diagnosis (24.6% vs 9.1% at relapse/progression), whereas *ACVR1* mutations were identified in 27.3% of tumors at relapse/progression compared to 10.3% at primary diagnosis. Four of the five detected *BRAF* V600E mutations were found in relapsed/progressive tumors (12.1% vs 0.8% at primary diagnosis). With regard to metastatic status, no clear differences in alteration frequencies were observed. Tumors of patients with M0 situation (*n* = 123) showed a slightly lower frequency of ALT positivity (27.6%) compared to tumors of patients with metastatic disease (any M+, *n* = 14; 50% ALT positive; *p* = 0.12), as well as more rarely alterations in the MAPK pathway (13.0% in M0 versus 35.7% in M+; *p* = 0.04) and in genes of cell cycle control (11.4% in M0 versus 28.6% in M+; *p* = 0.09). The cohort included tumors taken both before and after initiation of antitumor treatment, including radiotherapy. Even though alteration frequencies did also not obviously vary between radiotherapy-naїve (*n* = 130) and radiotherapy-exposed (*n* = 29) tumors, alterations in *PDGFRA* and *ATRX* occurred more often in tumors taken before radiotherapy (23.8% *PDGFRA*-altered and 20.8% *ATRX*-altered) compared to tumors taken after radiotherapy (10.3% *PDGFRA*-altered; *p* = 0.14 and 10.3% *ATRX*-altered; *p* = 0.29). On the other hand, there was a significant difference in the frequency of alterations in cell cycle control, which were found in 9.2% of tumors taken before and in 27.6% of tumors taken after radiotherapy (*p* = 0.01).

Especially with regard to the unfavorable prognosis of DMG patients, the identification of actionable targets in the respective tumors is essential. The analysis of the current cohort revealed genetic alterations that were considered as a potential target for targeted therapy in 131 tumors (80.9%), with alterations in e.g. *PDGFRA*, *FGFR1, EGFR* and *MET*, *KRAS*, *BRAF*, *PIK3CA* and *CDK4* being considered as priority targets. Together, alterations with a priority of ‘very high’ or ‘high’ according to the INFORM scoring system [51] were detected in 40 tumors (24.7%).

The median tumor mutational burden (TMB) was 0.49 somatic mutations per Megabase (Mb) (range 0.09–9.01). Thus, no tumor of the current cohort showed a hypermutator phenotype with a cut-off being set at TMB > 10 somatic mutations per Mb [6].

In 46 tumors (28.4%), a positive genomic signature for alternative lengthening of telomeres (ALT) was observed. Half of these tumors harbored *ATRX* alterations (23/30 *ATRX*-altered tumors).

Likely pathogenic germline alterations, which were considered to be potentially relevant for the current tumor disease were identified in six cases, namely affecting the genes *CHEK2* (*n* = 2), *PALB2*, *SLX4*, *MUTYH*, *NBN* (each *n* = 1). Whether these are truly playing a tumor driver role in this context, however, remains an open question. According to the information provided in the eCRF none of these alterations were known before in the respective patients’ medical history.

### Characteristics of specific subgroups

The most frequent tumor localization after pons/brainstem in the current cohort was the thalamus (*n* = 19). Median age at primary diagnosis of patients suffering from a thalamic DMG was slightly older compared to the whole cohort at 11 years (range 3–17 years). Molecularly, thalamic DMG were assigned to the DMG_K27 cohort in 17 tumors, with 14 of these harboring an H3.3 K27M mutation, whereas two tumors belonged to the DMG_EGFR subgroup. Almost half of the thalamic tumors had a positive genetic signature for ALT and notably, all three tumors with an *EZHIP* overexpression were located in the thalamus. Median overall survival (OS) of the current episode for patients with thalamic DMG was 14.0 months (range 4.5–31.8 months) with five patients being alive at last follow-up (no follow-up data available for two patients).

The nine patients with DMG located in the spinal cord were even older at primary diagnosis (median age 13 years, range 5–16 years). These tumors all belonged to the molecular DMG_K27 subgroup featuring the typical K27M mutation in histone H3.3. All patients of this group died of disease after a median OS of the current episode of 8.1 months (range 2.2–65.1 months).

One further specific molecular subgroup are the 21 tumors harboring a histone H3.1 K27M mutation. The respective patients were slightly younger at primary diagnosis compared to the whole cohort with a median age of 5 years (range 3–15 years). All except one patient were enrolled at primary diagnosis or with refractory/progressive disease. Most of these tumors were located in the pons or brainstem (*n* = 16; 76.2%). Median OS from primary diagnosis for this subgroup was 16.5 months (range 0.1–26.6 months) and three patients were alive at last follow-up. Notably, 76.2% of the H3.1 mutated tumors featured a concomitant *ACVR1* mutation, *BCOR* alterations were identified in 6 tumors (29%) and all tumors of this subgroup were negative for the ALT signature as well as for *ATRX* alterations.

Another interesting subgroup are tumors with alterations in the MAPK pathway (*n* = 27). Median age at primary diagnosis for these patients was 11 years (range 3–17 years) and median OS of the current episode 15.8 months (range 1.7–65.1 months). An H3.3 K27M mutation was detected in 25 tumors and H3.1 K27M mutation in one tumor. Within this group of MAPK-altered DMG, five tumors harbored a *BRAF* V600E mutation, which is a typical characteristic for lower-grade gliomas. One patient from each of these five patients was enrolled in the INFORM registry at primary diagnosis, first, second and third relapse, respectively, or due to refractory disease. Tumor localization was in the brainstem in one patient and the thalamus in two patients (no clear localization in two patients) and two patients had metastatic disease at the time point of INFORM enrollment. Molecularly, all five tumors were assigned to the DMG_K27 subgroup by methylation analysis and all harbored an H3.3 K27M mutation. One of the tumors featured an ALT-positive status and a potential pathogenic germline alteration was identified in two of these five patients (namely *CHEK2* and *SLX4*). After molecular analysis of the tumors through the INFORM pipeline, three patients received radiotherapy in combination with temozolomide (plus valproic acid in one patient). Two of these patients were treated with trametinib plus dabrafenib in addition. One patient received vemurafenib, cyclophosphamide and chloroquine (no data on treatment available for one patient). Tumor progression occurred in three patients after a median of 24.9 months (range 6.3–25.2 months) after diagnosis of the current episode (no data on progression available for two patients). Four patients died of the disease with a median survival of 14.6 months (1.7–37.4 months) after diagnosis of the current episode and 37.5 months (13.4–72.4 months) after primary diagnosis. One patient was alive at the last follow-up 24.9 months after diagnosis of the current episode and 28.1 months after primary diagnosis.

For the tumors assigned to the DMG_EGFR subgroup (*n* = 5), as well as the tumors with *EZHIP* overexpression or *EZH2* mutation (*n* = 4) and the two tumors without histone 3 K27 mutation or *EZHIP*/*EZH2* alteration within the DMG_K27 subgroup, small numbers hindered to make any reasonable comparisons.

### Gene expression analysis

**Fig. 3:**
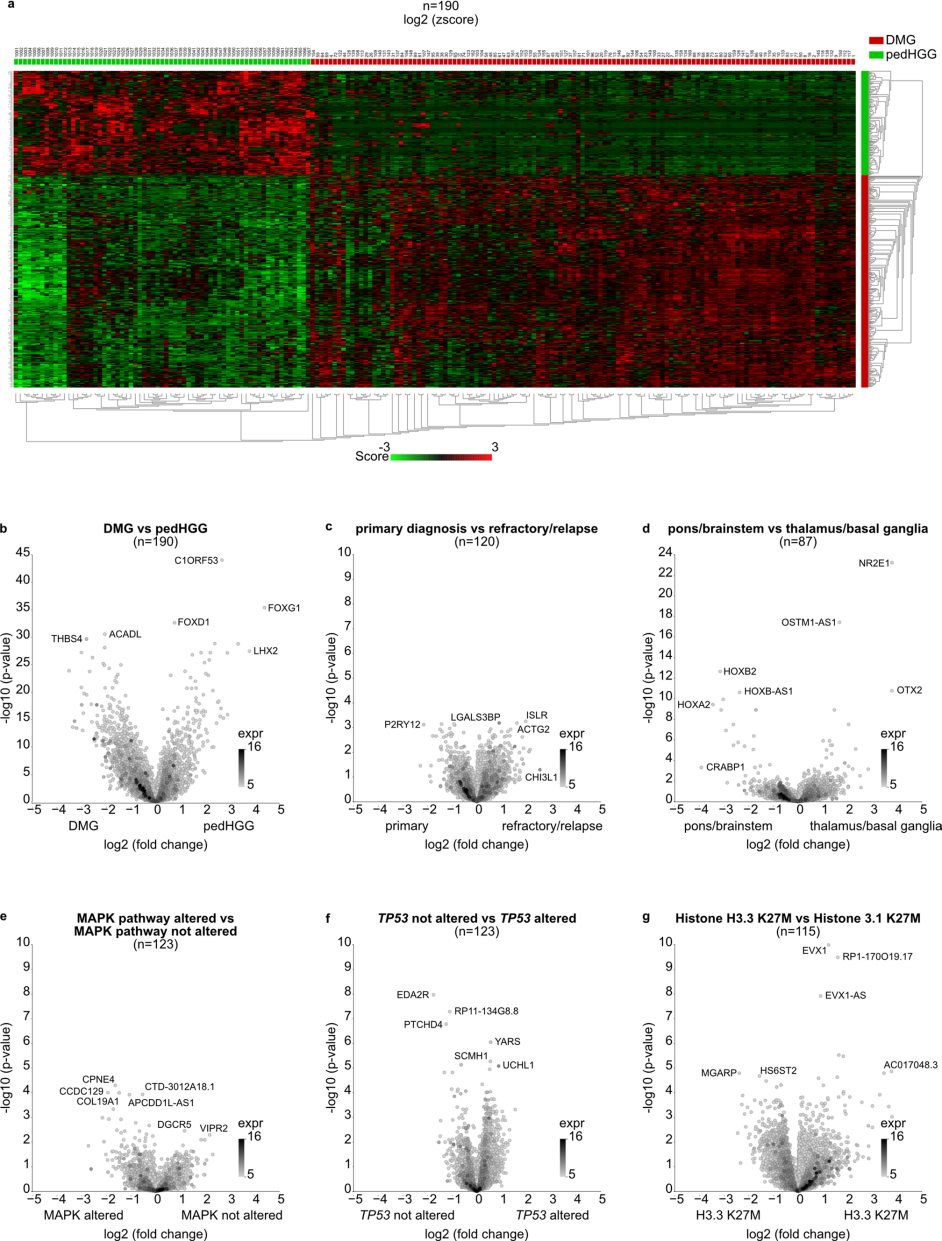
Differential gene expression comparing DMG and other pedHGG as well as several subgroups within the DMG cohort. **a** Heatmap with 500 most differentially expressed genes comparing 123 DMG and 67 non-midline, H3-wildtype pedHGG tumors shows separation of the two groups. For volcano plots, genes with high fold changes (log2 fold change; *x*-axis) and high statistical significance (− log10 of *p*-value; *y*-axis) are illustrated. **b** Volcano plot illustrating the most differentially expressed genes in DMG versus pedHGG. Volcano plots **c**–**g** comparing differential gene expression between subgroups, based on **c** current episode (primary diagnosis vs refractory/relapsed disease); **d** tumor localization (pons/brainstem vs thalamus); **e** MAPK pathway alteration status (alteration in MAPK pathway vs no alteration in MAPK pathway); **f** *TP53* alteration status (*TP53*-altered vs *TP53*-wildtype); **g** Histone H3 mutation status (Histone H3.1 K27M mutation vs Histone H3.3 K27M mutation). *DMG* diffuse midline glioma, *pedHGG* pediatric-type high-grade glioma, *expr* expression

Gene expression profiling based on RNA sequencing was available for 123/162 DMG. Comparison of differentially expressed genes between these samples of the current DMG cohort and 67 samples of non-DMG, histone 3 wild-type pedHGG patients from the INFORM registry showed a clear separation of the two groups based on *z*-Scores (Fig. 3a, b). The 500 most differentially expressed genes included exemplarily *NTRK2* (Supplementary Fig. 2a).

Furthermore, different subgroups within the DMG cohort were investigated and compared: current episode (primary diagnosis versus refractory/relapsed disease; Fig. 3c), tumor localization (pons/brainstem versus thalamus; Fig. 3d), MAPK pathway alteration status (MAPK pathway altered versus no MAPK pathway alteration; Fig. 3e), *TP53* alteration status (*TP53*-mutated versus *TP53*-wildtype; Fig. 3f) and Histone H3 mutation status (Histone H3.1 K27M mutation vs Histone H3.3 K27M mutation; Fig. 3g). Regarding tumor localization differentially expressed genes included *OTX2* (Supplementary Fig. 2b). Expression of *ALK*, *SETD2*, *ACVR1B* and *RAF1* (Supplementary Fig. 2c–f) as some examples was lower in tumors harboring a Histone H3.1 K27M mutation compared to tumors with Histone H3.3 mutation.

Gene set enrichment analysis applying the ‘oncogenic signature gene sets’ of the Human MSigDB Collections revealed enrichment in DMG compared to pedHGG of genes regulated upon expression changes of *PTEN*, *KRAS*, *MYC* and *ATM* as some examples (Supplementary Fig. 3a–d, Supplementary Table 1).

### Treatment data

Data on treatment of the current episode were available for 153 patients (94.4%), previous treatment (if applicable) for 35 patients and outcome data for 145 patients (89.5%; not available *n* = 17), respectively.

The majority of patients enrolled at primary diagnosis (*n* = 126, including 3 patients with no treatment data available) were treated with radiotherapy (*n* = 105; 85.4%), with nine of these patients receiving no additional systemic therapy. Besides the widely applied systemic therapy with temozolomide and/or valproic acid (*n* = 89), other chemotherapeutic agents were applied in 11 patients, tyrosine kinase inhibitors in 40 patients, other targeted therapeutic agents in six patients, immunotherapy in two patients and other treatments such as chloroquine or methadone in eight patients (several patients received multiple treatments). A total of 55 patients enrolled at primary diagnosis (44.7%) received at least one further therapeutic element in addition or instead of the standard of care treatment (radiotherapy plus temozolomide). No further treatment after surgery was applied to ten patients.

Regarding treatment regimen applied for the current episode in patients with relapsed/refractory disease (*n* = 33, including four patients with no data on current treatment available), 14 patients were treated with radiotherapy (42.4%); of these nine received re-radiotherapy. Systemic therapy consisted of temozolomide (with or without valproic acid) in nine patients, other chemotherapeutic therapies in nine patients, tyrosine kinase inhibitors in 17 patients, other targeted therapies in four patients, immunotherapy in one and other therapies in three patients. Several patients were treated with different approaches and three patients did not receive any further treatment. Treatment of previous disease episodes in this group of patients included radiotherapy or various systemic therapy approaches, respectively, in 28/33 patients each. Eight patients had received tyrosine kinase inhibitors (*n* = 7) or immunotherapy (*n* = 1). Three patients had been treated with radiotherapy only and two other patients enrolled at first relapse were treatment-naїve besides surgery.

### Targeted therapy based on molecular profiling

A total of 64 patients (41.8% of patients with data on treatment available) were treated with targeted therapies (TT; including immunotherapy and tumor-specific vaccination) after enrollment in the INFORM registry. A list of all applied TTs is provided as Supplementary Table 2. Most of these patients received one TT (*n* = 32) or two different TTs (*n* = 21). Three TTs were applied in six patients, four TTs in three patients and treatment included five different TTs in two patients. In total, 114 treatments with TT were initiated in the whole cohort. In 41 patients treated with a total of 51 TTs, the TT was considered matching to respective genetic alterations identified in the INFORM analysis (ten patients were treated with two matching TTs). Thirty of these TTs were directly targeting the respective alteration, whereas the affected pathway was targeted further downstream with 21 TTs. The respective genetic alterations affected the following genes (in some patients several TTs were applied targeting the same alteration): *PDGFRA* (*n* = 8), *EGFR* (*n* = 2), *MET* (*n* = 5), *FGFR1* (*n* = 3), *NRAS/KRAS* (*n* = 2), *BRAF* (*n* = 4), *NF1* (*n* = 4), *MAPK3* (*n* = 1), *PIK3CA* (*n* = 13), *PTEN* (*n* = 1), *CDK4* (*n* = 1), *CCND1* (*n* = 1), *NTRK* (*n* = 1), *TSC1* (*n* = 1) and H3 vaccine (*n* = 1). Notably, alterations of *KDR/KIT* (concomitant with *PDGFRA*) were additionally targeted in four patients with tumors harboring *PDGFRA/KDR/KIT* co-amplification.

The most frequently applied TT was ONC201, a dopamine receptor 2 antagonist, which was applied in 19 patients. However, this was not considered to be a particular match to a corresponding alteration due to uncertainties about biomarkers/mechanism-of-action for this compound. The mTOR inhibitor everolimus was part of the therapeutic concept in 13 patients, with genetic alterations in the mTOR pathway being identified in the tumors of eight of these patients. At a similar frequency, trametinib (MEK inhibitor), was used in 12 patients—in two-third of cases (*n* = 8) in line with alterations affecting the MAPK pathway. Sirolimus (mTOR inhibitor) and paxalisib (PI3K inhibitor) were each prescribed in seven patients. Eight and seven patients, respectively, received bevacizumab (VEGF inhibitor) and nivolumab (checkpoint inhibitor) without a corresponding alteration identified in the respective tumor.

### Outcome

Follow-up data on the further clinical course after the current episode with enrollment in the INFORM registry were available for 145 patients (no follow-up data available for 17 patients, two patients excluded from respective analysis due to missing date of primary diagnosis, one patient due to missing date of diagnosis of current episode). Three patients enrolled at primary diagnosis were lost to follow-up less than 6 months after INFORM analysis (still included in survival analysis).

Disease progression after enrollment in the INFORM registry was documented in 103 patients, with a median time to progression of 7.4 months (range 1.8–28.7) from diagnosis of the current event (data on progression missing for 16 patients, five patients died before the first response assessment scheduled 3 months after INFORM analysis). In total, 124/145 patients deceased from the disease during the follow-up period. Median overall-survival (OS) time of these patients from primary diagnosis was 12.9 months (range 1.2–98.5) and 11.0 months (range 1.2–65.1) from diagnosis of the event leading to the enrollment in the INFORM registry. Twenty-one patients were alive at last follow-up, with a median time from diagnosis of the current event of 15.5 months (range 0.1–29.2).

Outcome analysis using Kaplan–Meier estimator revealed a 2-year overall survival (2y-OS) rate for the whole cohort of 10.5% from diagnosis of the current episode and 16.9% from primary diagnosis.

Seventeen patients survived more than 24 months from primary diagnosis (median OS from primary diagnosis 32.7 months, range 26.5–98.5). Seven of these patients were enrolled in INFORM at primary diagnosis, four patients each at first and second or further relapse, whereas refractory/progressive disease under first-line treatment lead to enrollment in INFORM in two patients. One of these patients was primarily diagnosed with an oligoastrocytoma °III more than 7 years preceding the current diagnosis of high-grade glioma. For another patient diagnosis of tuberous sclerosis with giant cell astrocytoma was documented in the medical history—however, no respective germline alteration was identified in the INFORM analysis. A potentially pathogenic germline alteration was identified in three patients of this group. Molecularly, 16 tumors were assigned to the DMG_K27 subgroup with 15 tumors harboring a histone 3 K27M mutation. Notably, the tumors of four longer-term survivors harbored a *BRAF* V600E mutation.

**Fig. 4:**
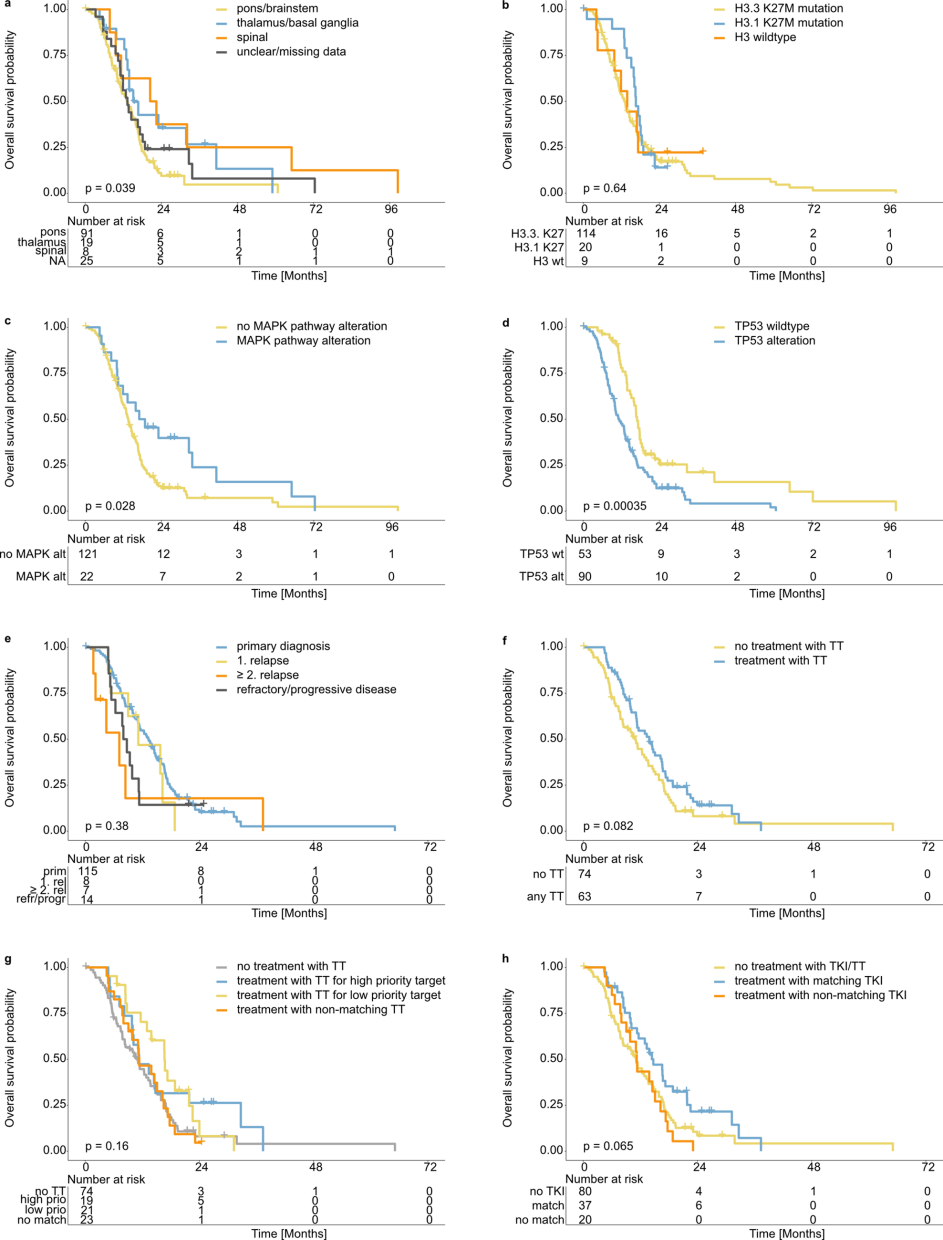
Outcome analysis of 145 DMG patients. **a** Overall survival (OS) according to tumor localization (from primary diagnosis); **b** OS according to Histone 3 K27M mutation status (from primary diagnosis); **c** OS according to MAPK pathway alteration (from primary diagnosis); **d** OS according to *TP53* alteration status (from primary diagnosis); **e** OS according to disease episode (from diagnosis of current episode); **f** OS according to general treatment with targeted therapy (from diagnosis of current episode); **g** OS according to treatment with targeted therapy based on respective target identified (from diagnosis of current episode); **h** OS according to treatment with tyrosine kinase inhibitor based on respective target (from diagnosis of current episode). *NA* not applicable, *H3.1* histone 3.1, *H3.3* histone 3.3, *wt* wildtype, *TT* targeted therapy, *TKI* tyrosine kinase inhibitor

For survival analysis from primary diagnosis there was a slightly better outcome observed for patients with tumor localized in the spinal cord by univariate analysis (HR 0.396, *p*-value 0.026, 95% CI 0.175–0.897) and thalamus/basal ganglia (HR 0.555, *p*-value 0.046, 95% CI 0.311–0.991) compared to pons/brainstem (Fig. 4a). Notably, alterations in the MAPK pathway as well as *TP53* alterations were associated with a significant difference in survival (Fig. 4c + d, Supplementary Table 3) whereas patients’ outcome did not differ significantly according to presence or type of histone H3 K27M mutation (Fig. 4b). However, in multivariate analysis only *TP53* status was confirmed as a risk factor (*TP53* alteration HR 1.888, *p*-value 0.0015, 95% CI 1.275–2.796; MAPK alteration HR 0.637, *p*-value 0.107. 95% CI 0.368–1.103; spinal localization HR 0.419, *p*-value 0.035, 95% CI 0.186–0.943). This is likely due to an interrelationship between the two variables—with MAPK-altered tumors being more likely to be *TP53*-wildtype. Investigating outcome according to MAPK pathway alteration status only in patients with *TP53*-wildtype tumors (*n* = 53) confirmed the difference in survival in association with the presence of MAPK pathway alteration (Supplementary Fig. 4a; HR 0.395, *p*-value 0.03, 95% CI 0.170–0.922).

For the evaluation of patients’ outcome according to the treatment applied survival analysis was performed from the diagnosis of the current event. No significant difference was observed according to ‘type’ of current episode (primary diagnosis vs relapsed/refractory/progressive disease, Fig. 4e, Supplementary Table 3) as well as stratified by treatment with targeted therapy in general or based on priority of respective targets identified in the INFORM analysis (Fig. 4f + g, Supplementary Table 3). However, when comparing treatment with no or not matching TTs to treatment with matching TTs a significant difference in patients’ outcome was observed (Supplementary Fig. 4b, Supplementary Table 3). The same holds true when comparing survival from primary diagnosis in patients receiving standard-of-care treatment only, namely radiotherapy plus temozolomid ± valproic acid, and patients receiving matching targeted therapy anytime during the clinical course (Supplementary Fig. 4, Supplementary Table 3). For the treatment with tyrosine kinase inhibitors (Fig. 4h, regardless of other TTs) as well as for the treatment with ONC201 (Supplementary Fig. 4d, Supplementary Table 3) there seems to be a trend towards better outcome. To investigate whether the observed difference in outcome according to MAPK pathway alteration status was related to the treatment with respective TT, survival analysis was performed for the group of patients with MAPK-altered tumors only (*n* = 21). Also in this group, no significant difference in outcome was observed according to treatment with TT (matching/not matching to the MAPK alteration) compared to no treatment with TT (Supplementary Fig. 4e; Supplementary Table 3).

## Discussion

During the last 15–20 years, knowledge on the molecular characteristics of pediatric DMG has grown considerably [10, 14, 33] and various treatment strategies for this devastating disease have been investigated [45]. However, no approach has yet been able to alter the very unfavorable prognosis of the affected patients. Thus, there is still an urgent clinical need to identify and implement new therapeutic options for DMG patients. Prospective molecular characterization of the tumors through the INFORM pipeline allows for a rapid integration of genetic findings in clinical treatment decisions, which is especially crucial for DMG patients. The current report on 162 primary and relapsed/progressive DMG patients of the INFORM registry combines comprehensive molecular characterization of the respective tumors with clinical data on the patients’ treatment and outcomes. By describing the process starting from the results of molecular analysis up to the individual application of targeted therapies in clinical care, this study contributes to a better understanding of the feasibility and implementation of targeted therapies in DMG patients in a real-world setting.

Molecular characterization of the DMG cohort presented here, revealed a landscape of alterations similar to previous reports [33], e.g. for typically and frequently altered genes like *TP53, ATRX, ACVR1, PDGFRA, NF1, PIK3CA, MYC/MYCN* and *EGFR*. Looking at the distribution of alterations in specific subgroups, differences in the frequency can be observed. *PDGFRA* alterations were reported to be found more often in tumors of the pons, whereas alterations of *FGFR1* more frequently in thalamic tumors [19, 21]. In our cohort, 60% of *PDGFRA*-altered tumors were located in pons/brainstem. For *FGFR1,* the spatial distribution of the respective tumors was more even with 27% of *FGFR1*-altered tumors being located in each the pons/brainstem and spinal cord and 36% in the thalamus. Specific characteristics attributed to the subgroup of H3.1 K27-mutated tumors, like localization in the pons, younger age and association with *ACVR1* mutation [10, 46] have been reproduced in the current cohort. The reported better outcome of patients with tumors harboring H3.1 K27M mutation [10], however, was not observed in our cohort (Fig. 4b). Reasons for this discrepancy might include limited follow-up data beyond 2 years after INFORM analysis (e.g. three patients with H3.1-mutated tumors were still alive > 20 months after primary diagnosis without further follow-up data available).

Furthermore, alterations affecting the PI3K/mTOR pathway were described to occur more frequently in H3.1 K27-mutated tumors [33]. Accordingly, in our cohort concomitant *PIK3CA* or *PIK3R1* alterations were identified more often in H3.1 K27-mutated tumors compared to H3.3 K27-mutated tumors (40% in H3.1 vs 20.9% in H3.3).

The highest frequency of alterations in the MAPK pathway has previously been described to occur in H3-wildtype pedHGG [33]. The discrepancy in our cohort with all MAPK-altered tumors harboring an H3 K27M mutation, might be attributed to the selection in the current cohort, excluding other HGG entities like PXA and localizations other than midline structures. In line with previous reports describing a higher frequency of MAPK-altered tumors in the cohort of DMG long-term survivors [21, 40], seven of seventeen tumors of patients surviving more than 24 months after primary diagnosis harbored an alteration in the MAPK pathway (BRAF *n* = 4, KRAS *n* = 2, NF1 *n* = 1). In addition, OS in our cohort was significantly better in patients with tumors harboring MAPK alterations.

A recent publication describes a subtype within DMG characterized by H3 K37M and *BRAF*/*FGFR1* co-alteration [3] with distinct clinical and genetic features. In contrast to this study by Auffret et al., in our cohort based on DNA methylation analysis the tumors with H3 K27M and concomitant *BRAF* V600E or *FGFR1* mutation did not form a separate cluster in the t-SNE but were spread across the DMG cluster (Supplementary Fig. 5). In line with the study by Auffret et al., a trend towards better survival could be observed within the group of H3 K7M-mutated DMG for the patients with co-alteration in *BRAF*/*FGFR1* (Supplementary Fig. 4f). However, small patient numbers do not allow for conclusive analysis.

The observation from the current cohort of a worse outcome for patients with *TP53*-mutated DMG is also consistent with previous reports [50]. Mutations in this key tumor suppressor may promote aggressive tumor behavior and reduce responsiveness to standard treatments, including radiotherapy.

A recent publication by Stegat et al. described two DMG subgroups based on methylation clustering with distinct clinical and molecular features [43]. Whereas group DMG-A was characterized by e.g. older age and better outcome of the patients as well as higher frequency of MAPK pathway alterations in the tumors, patients in the DMG-B group were younger at diagnosis, had worse survival and a high proportion of the tumors carried *TP53* alterations. Even though, in this study the impact of specific parameters on patients’ outcome was dependent on the subgroup affiliation and we did not see a clear separation of the two described subgroups, we consider our findings from the current cohort to be largely concordant with the above-mentioned distinction.

Mateos et al. [34] recently published a study on germline alterations in DMG, which also included patients from the INFORM registry and therefore partially overlapped with the current cohort. In the cohort by Mateos pathogenic or likely pathogenic germline alterations were described at a slightly higher frequency of 7.5% of patients, compared to 3.7% in the cohort presented here. As in Mateos’ cohort, altered genes in our cohort included *CHEK2*, *PALB2* and *SLX4*. With regard to concomitant somatic mutations, these were found in the same frequently altered pathways as in the counterparts without germline alterations (in parentheses the respective genes affected by germline alteration in the current cohort are provided), namely *TP53* (*MUTYH* and *NBN* germline mutation), *ATRX* (*PALB2* and *CHEK2* germline mutation), *PPM1D* (*PALB2* germline mutation), *BRAF* (*CHEK2* and *SLX4* germline mutation), *NF1* (*CHEK2* germline mutation) and *PDGFRA* (*PALB2* germline mutation). Notably, none of the six tumors of patients with germline alterations showed somatic alterations in the PI3K-mTOR pathway—an observation which was shared by Mateos et al.

Treatment of DMG patients is a challenge for neuro-oncologists due to the lack of sustainable efficacy of the common treatment regimen. According to the recently published “European standard clinical practice recommendations for paediatric high-grade gliomas” [45] radiotherapy constitutes the core component of treatment for DMG patients. Regarding a specific chemotherapy regimen, there is no clear consensus and evidence. The role of temozolomide is overall controversial—though the better tolerability compared to other chemotherapeutic regimen underpins that temozolomide is typically considered standard of care [45]. The opinions among (pediatric) neuro-oncologists regarding the most relevant treatment regimen are heterogeneous, especially in the situation of relapse or progression [17, 38]. Various treatment options, including chemotherapeutics, targeted therapies and immunotherapy are discussed elsewhere [1, 28, 29]. Some small case series with non-prospective designs report on the limited clinical impact of targeted therapy based on individual molecular characteristics of the respective tumors [22]. There are also several clinical trials ongoing investigating new treatment approaches, such as CAR-T cells targeting GD2 (NCT05544526), vaccine and immunotherapeutic strategies (NCT06305910; NCT04943848) and different targeted small molecules (NCT05099003; NCT05843253). The ambiguity regarding the most effective treatment regimen is likewise reflected in the various different therapeutic strategies applied in the current cohort.

During the last years, ONC201, a dopamine receptor D2 (DRD2) antagonist and caseinolytic protease P (ClpP) agonist, has gained interest as a potential new therapeutic drug for H3 K27M-mutated DMG. In the cohort presented here, ONC201 was the most frequently applied compound (other than temozolomide) with 11.7% of patients (19/162) being treated with ONC201 after enrollment in the INFORM registry. Mechanistically, the mode of action of ONC201 in DMG is broadly considered to be by impairing metabolic and epigenetic functions and interfering with the effects of H3K27me3 loss [49]. Several early phase clinical trials, including a phase I trial in pediatric DMG patients and compassionate use/expanded access programs have been conducted in primary as well as recurrent DMG patients [2, 13, 15, 20]. However, evaluation of efficacy is still equivocal, with only anecdotal response of single patients [23, 24] and therefore, to date the role of ONC201 in DMG treatment remains controversial. In our cohort, no significant difference in patients’ outcome was observed based on treatment with ONC201. However, small patient numbers and concomitant medication with other therapeutic agents in our cohort limited the interpretation of survival analysis with regard to this treatment.

In the cohort presented here, targetable alterations were identified in a substantial proportion of tumors, with targets attributed to have a ‘very high’ or ‘high’ priority according to the INFORM priority algorithm being found in about one quarter of patients. Notably, in only half of the patients being treated with matching TT, the respective genetic target in the tumor was given a priority of ‘very high’ (6/40) or ‘high’ (13/40). These alterations included genetic findings in *PDGFRA*, *EGFR*, *FGFR1*, *MET*, *NRAS/KRAS*, *BRAF* and *NF1*. Previous analyses of data from the INFORM registry showed a trend towards better event-free survival in patients with ‘very high’ priority level target in the tumor receiving matched TT [48]. However, a subsequent evaluation suggested that this benefit from matching TT for respective genetic alterations may be restricted to ALK, NTRK and BRAF inhibitors [26]. In line with these previous findings, we did not observe a difference in OS for patients treated with TT matching to a ‘high’ or ‘very high’ priority target compared to patients treated with TT matching to a target with lower priority (data not shown).

Comparing treatment with TT at primary diagnosis versus in the situation of relapse/progressive disease, 57.6% (19/33) of patients enrolled at relapse/progression received treatment with TT after the INFORM analysis, compared with 43/126 (34.1%) patients analyzed at primary diagnosis. For the patients with relapsed/progressive disease, the previous medical history reported treatment with TT in 21.2% of patients. Taken together, these numbers illustrate that prospective molecular characterization of the tumor can have an impact on treatment decisions, especially in the desperate situation of relapsed/progressive DMG.

There are several limitations of the study presented here. The interpretation of specific subgroup analyses, e.g. for gene expression profiling or outcome data, is impaired by small patient numbers. Due to the character of INFORM as a registry without intervention, there was a vast heterogeneity of treatment regimen applied for the enrolled patients. Confounding reasons might have influenced the decision on treatment, especially the application of TTs, e.g. whether the patient’s general condition was good enough for considering an experimental treatment. Therefore, detailed outcome analyses comparing specific treatment approaches were not feasible in the current cohort, also due to the partly incomplete documentation on treatment and follow-up. Prospective controlled clinical trials would be necessary for the evaluation of treatment efficacy.

In conclusion, the classification of pediatric DMG in different molecular subgroups was confirmed in the current study, further identifying typical genetic alterations that might in parts serve as targets for TT. We could demonstrate that the implementation of TT based on molecular characteristics of the tumor on the individual patient level was feasible and realized in one third of patients. However, treatment with single TT showed no impact on overall survival and therefore, new treatment approaches for DMG patients in order to achieve sustained improvement in patients’ outcome are still urgently needed.

## Supplementary Information

Below is the link to the electronic supplementary material.

Supplementary file1 **Supplementary Fig. 1**: CONSORT diagram showing selection of patients for this study from all patients enrolled in INFORM. HGG = high-grade glioma; DIPG = diffuse intrinsic pontine glioma; IDH1 = Isocitratdehydrogenase 1; H3 G34-mutated HGG = high-grade glioma harboring Histone 3 mutation at position G34; DMG K27-altered = diffuse midline glioma with alteration of Histone 3 at position K27 (TIFF 718 KB)

Supplementary file2 **Supplementary Fig. 2**: Violin plots showing gene expression of selected genes in different subgroups. a) *NTRK2* is higher expressed in the DMG cohort compared to other pedHGG tumors; b) *OTX2* shows a higher expression in DMG localized in the thalamus compared to tumors in brainstem/pons; expression of several genes is lower in tumors harboring histone H3.1 K27M mutation compared to tumors with Histone H3.3 K27M mutation including c) *ALK*, d) *SETD2,* e) *ACVR1B* and f) *RAF1*. DMG = diffuse midline glioma; pedHGG = pediatric-type high-grade glioma; Histone 3.3 K27M = Histone 3.3 K27M-mutation positive; H3.1 K27M = Histone 3.1 K27M-mutation positive (TIFF 1270 KB)

Supplementary file3 **Supplementary Fig. 3:** Gene set enrichment analysis. Enrichment of the following gene sets was observed in DMG compared to pedHGG: a) genes up-regulated upon knockdown of *PTEN*; b) genes up-regulated upon *KRAS* overexpression; c) genes down-regulated upon *MYC* overexpression; d) genes down-regulated upon knockdown of *ATM*. DMG = diffuse midline glioma; pedHGG = pediatric-type high-grade glioma (TIFF 2846 KB)

Supplementary file4 **Supplementary Fig. 4**: Outcome analysis. a) Overall survival (OS) according to MAPK pathway alteration in *TP53*-wildtype tumors (n = 53, from primary diagnosis); b) OS according to treatment with matching targeted therapy (n = 137, from diagnosis of current episode); c) OS according to treatment with standard of care (SOC, radiotherapy + temozolomide ± valproic acid) versus treatment with matching targeted therapy at any time (n = 81, from primary diagnosis); d) OS according to treatment with ONC201 (n = 137, from diagnosis of current episode); e) OS according to treatment with targeted therapy in tumors with MAPK pathway alteration (n = 21, from diagnosis of current episode); f) OS according to *BRAF*/*FGFR1* mutation in H3 K27M-mutated tumors (n = 134, from primary diagnosis) (TIFF 1131 KB)

Supplementary file5 **Supplementary Fig. 5:** Clustering based on whole-genome DNA methylation analysis visualized by t-stochastic neighbor embedding (t-SNE) combined with DMG_K27 samples of the reference cohort from Capper et al. [7]. DMG_K27 Capper = methylation class diffuse midline glioma with Histone 3 K27-alteration included in cohort from Capper; DMG_K27/K27-wt = methylation class diffuse midline glioma Histone 3-altered, without K27M-mutation; DMG_K27/H3.3 K27-mut = methylation class diffuse midline glioma with Histone 3.3 K27M-mutation; DMG_K27/H3.1 K27-mut = methylation class diffuse midline glioma with Histone 3.1 K27M-mutation; DMG_K27/EZHIP-EZH2 = methylation class diffuse midline glioma Histone 3-altered, with *EZHIP* overexpression or EZH2 alteration; DMG_EGFR/EGFR-alt = methylation class diffuse midline glioma *EGFR*-altered, with *EGFR* alteration; DMG_EGFR/EGFR-with = methylation class diffuse midline glioma *EGFR*-altered, without *EGFR* alteration; DMG_K27/FGFR1-BRAF = methylation class diffuse midline glioma Histone 3-altered with *BRAF* V600E or *FGFR1* mutation (TIFF 1125 KB)

Supplementary file6 **Supplementary Table 1**: Results of gene set enrichment analysis applying the ‘oncogenic signature gene sets’ of the Human MSigDB Collections. ES = enrichment score; NES = normalized enrichment score; FDR = false discovery rate; FWER = familywise-error rate; Rank at max = Position in ranked list with maximum enrichment score (DOCX 19 KB)

Supplementary file7 **Supplementary Table 2**: List of all applied targeted therapeutic agents (DOCX 12 KB)

Supplementary file8 **Supplementary Table 3**: Results of univariate analysis. Endpoint = overall survival. OS = overall survival; DMG_K27 = methylation class diffuse midline glioma Histone 3 K27-altered; DMG_EGFR = methylation class diffuse midline glioma *EGFR*-altered; H3.3 K27 = Histone 3.3 K27M-mutation positive; H3.1 K27 = Histone 3.1 K27M-mutation positive; H3 wt = Histone 3 wildtype; TT = targeted therapy; TKI = tyrosine kinase inhibitor; SOC = standard of care treatment with radiotherapy + temozolomide ± valproic acid (DOCX 15 KB)

## Data Availability

Data are provided within the manuscript or supplementary information files. Sequence data will be made available via the German Human Genome-Phenome Archive (GHGA, https://www.ghga.de/).
