# Supplementary figures and images for: Molecular characterization and clinical features of diffuse midline glioma in the pediatric precision oncology registry INFORM
Source: Acta Neuropathol. 2025 Oct 11;150(1):42. doi: 10.1007/s00401-025-02945-9 (PMC12515216; doi:10.1007/s00401-025-02945-9)

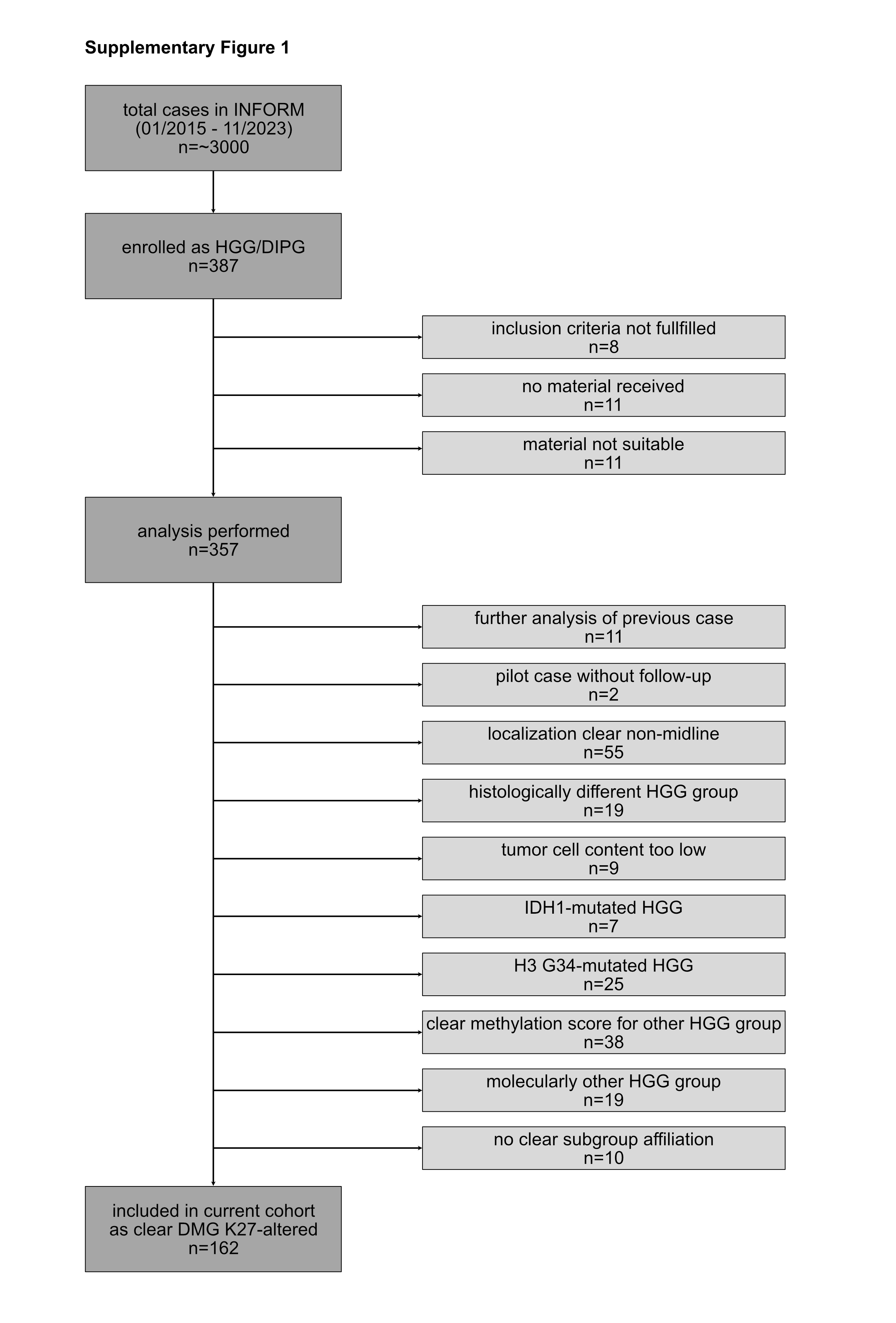

Supplement: Supplementary file 1 — Supplementary file1 Supplementary Fig. 1: CONSORT diagram showing selection of patients for this study from all patients enrolled in INFORM. HGG = high-grade glioma; DIPG = diffuse intrinsic pontine glioma; IDH1 = Isocitratdehydrogenase 1; H3 G34-mutated HGG = high-grade glioma harboring Histone 3 mutation at position G34; DMG K27-altered = diffuse midline glioma with alteration of Histone 3 at position K27 (TIFF 718 KB) [file 401_2025_2945_MOESM1_ESM.tiff]

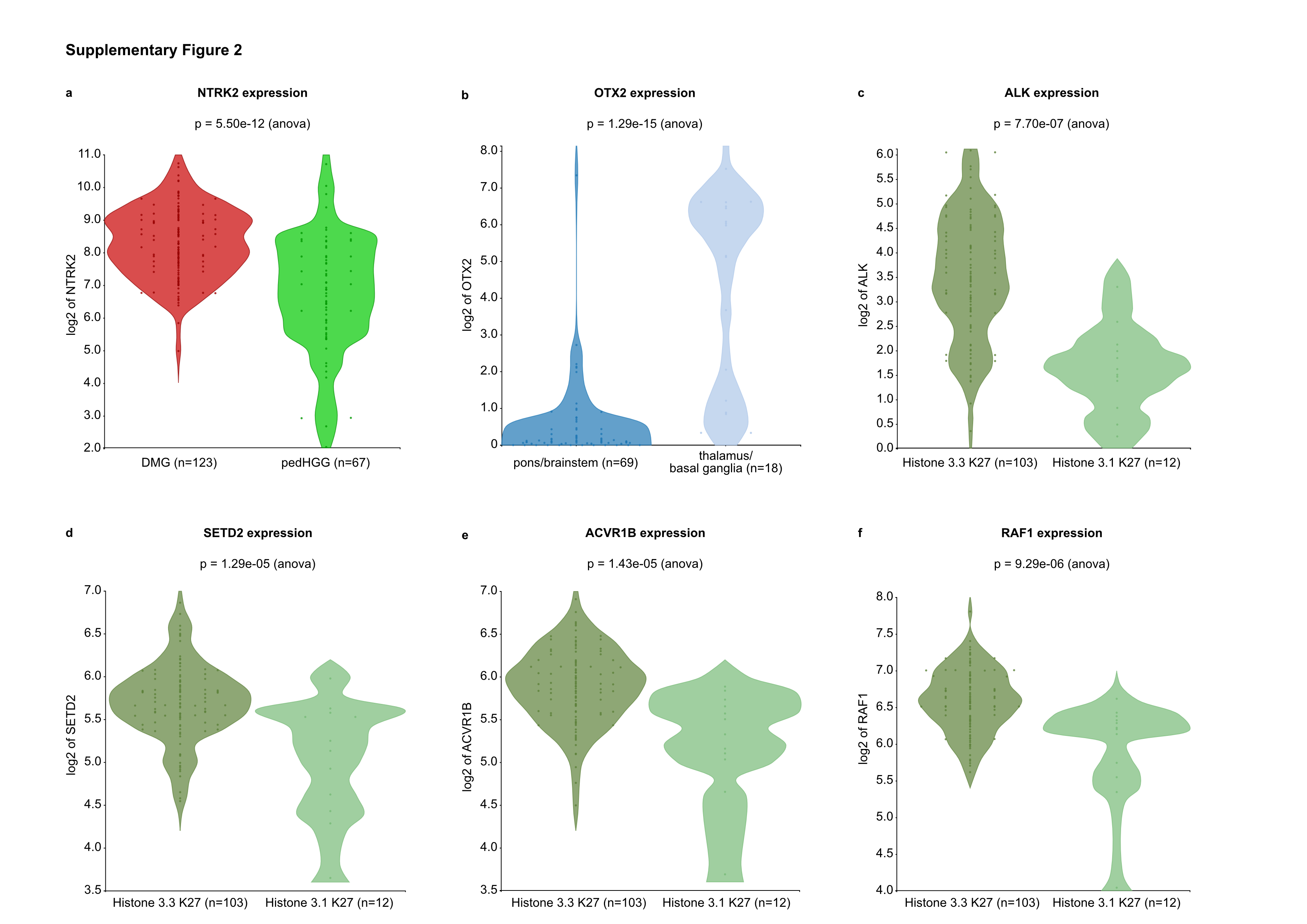

Supplement: Supplementary file 2 — Supplementary file2 Supplementary Fig. 2: Violin plots showing gene expression of selected genes in different subgroups. a) NTRK2 is higher expressed in the DMG cohort compared to other pedHGG tumors; b) OTX2 shows a higher expression in DMG localized in the thalamus compared to tumors in brainstem/pons; expression of several genes is lower in tumors harboring histone H3.1 K27M mutation compared to tumors with Histone H3.3 K27M mutation including c) ALK, d) SETD2, e) ACVR1B and f) RAF1. DMG = diffuse midline glioma; pedHGG = pediatric-type high-grade glioma; Histone 3.3 K27M = Histone 3.3 K27M-mutation positive; H3.1 K27M = Histone 3.1 K27M-mutation positive (TIFF 1270 KB) [file 401_2025_2945_MOESM2_ESM.tiff]

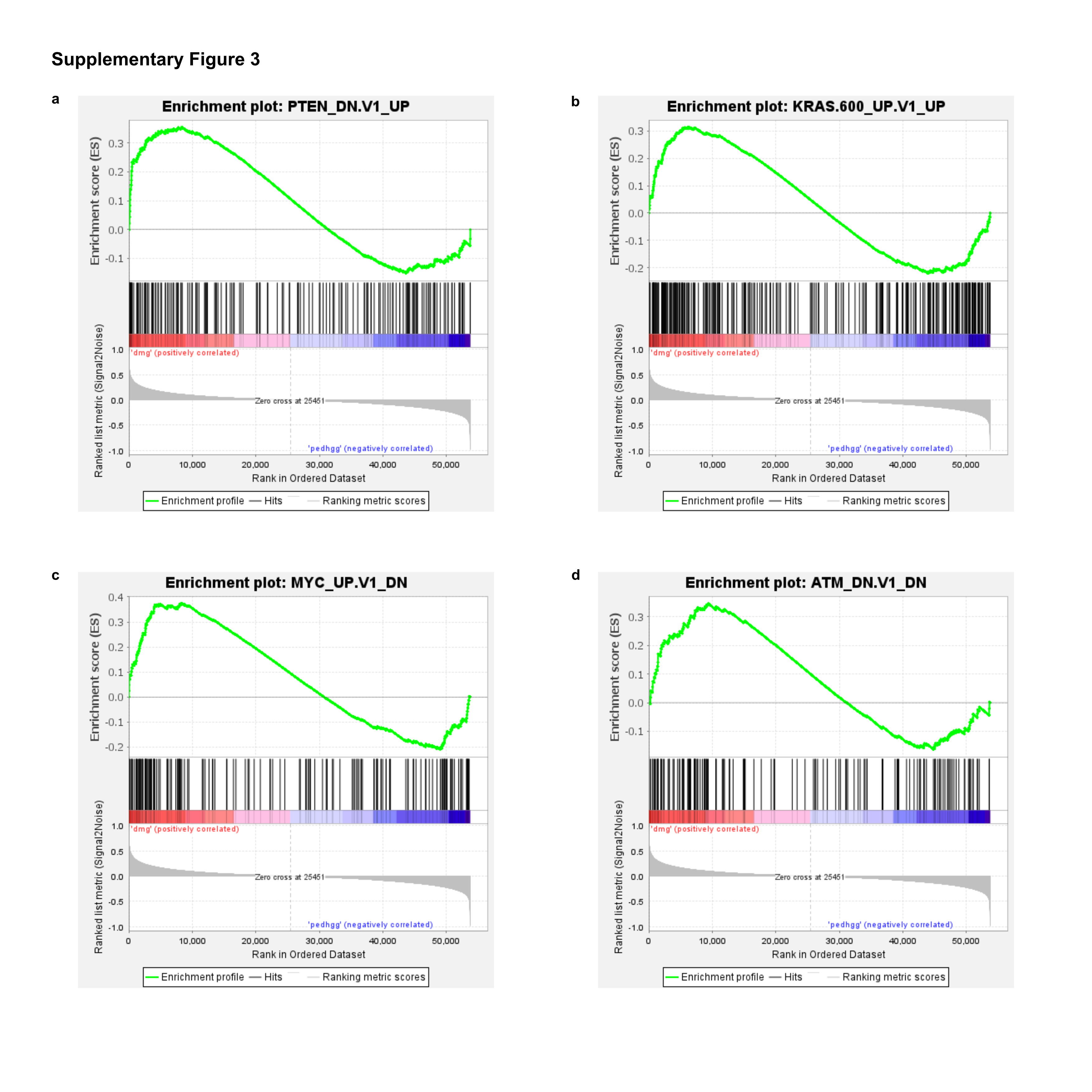

Supplement: Supplementary file 3 — Supplementary file3 Supplementary Fig. 3: Gene set enrichment analysis. Enrichment of the following gene sets was observed in DMG compared to pedHGG: a) genes up-regulated upon knockdown of PTEN; b) genes up-regulated upon KRAS overexpression; c) genes down-regulated upon MYC overexpression; d) genes down-regulated upon knockdown of ATM. DMG = diffuse midline glioma; pedHGG = pediatric-type high-grade glioma (TIFF 2846 KB) [file 401_2025_2945_MOESM3_ESM.tiff]

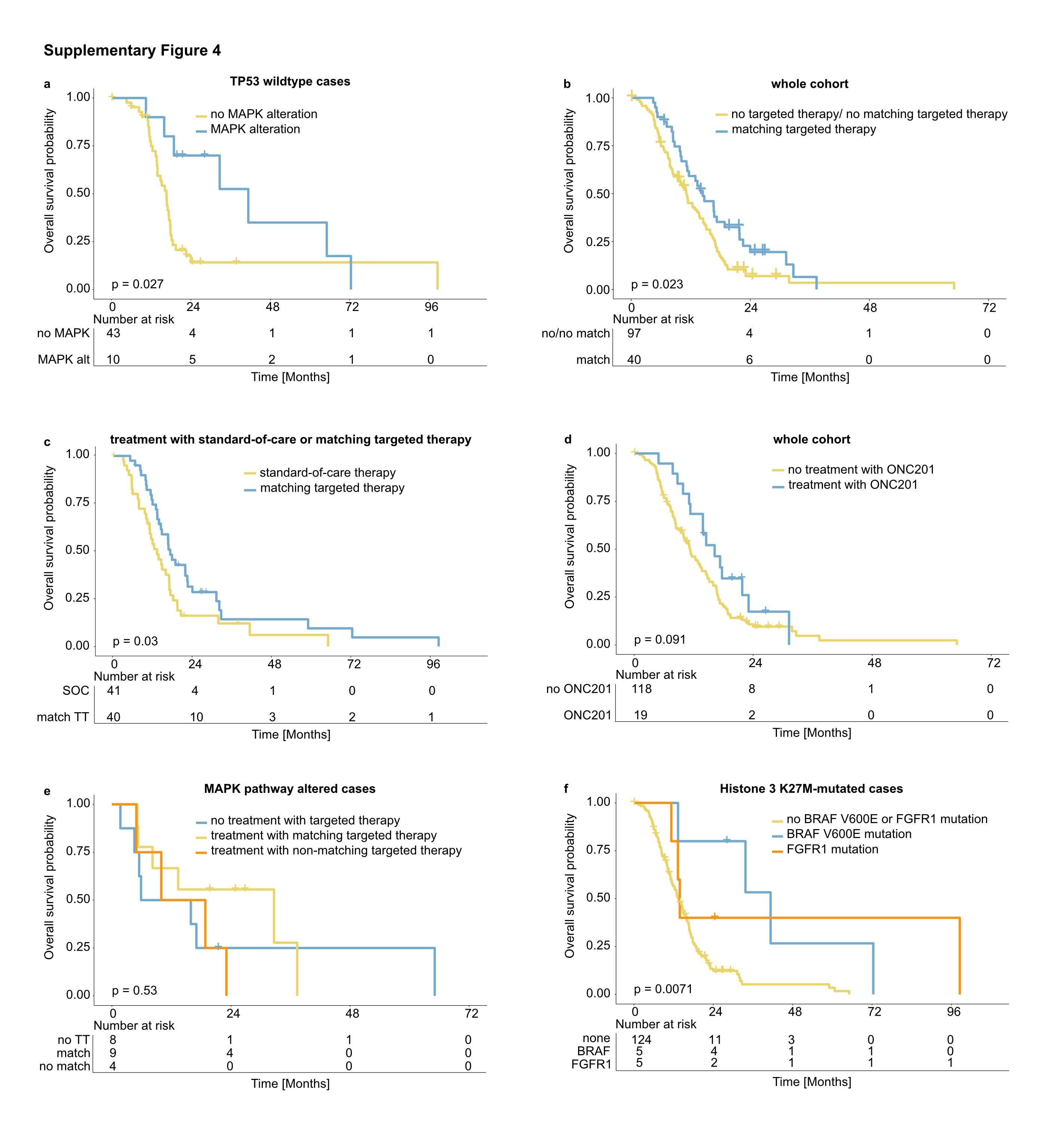

Supplement: Supplementary file 4 — Supplementary file4 Supplementary Fig. 4: Outcome analysis. a) Overall survival (OS) according to MAPK pathway alteration in TP53-wildtype tumors (n = 53, from primary diagnosis); b) OS according to treatment with matching targeted therapy (n = 137, from diagnosis of current episode); c) OS according to treatment with standard of care (SOC, radiotherapy + temozolomide ± valproic acid) versus treatment with matching targeted therapy at any time (n = 81, from primary diagnosis); d) OS according to treatment with ONC201 (n = 137, from diagnosis of current episode); e) OS according to treatment with targeted therapy in tumors with MAPK pathway alteration (n = 21, from diagnosis of current episode); f) OS according to BRAF/FGFR1 mutation in H3 K27M-mutated tumors (n = 134, from primary diagnosis) (TIFF 1131 KB) [file 401_2025_2945_MOESM4_ESM.tiff]

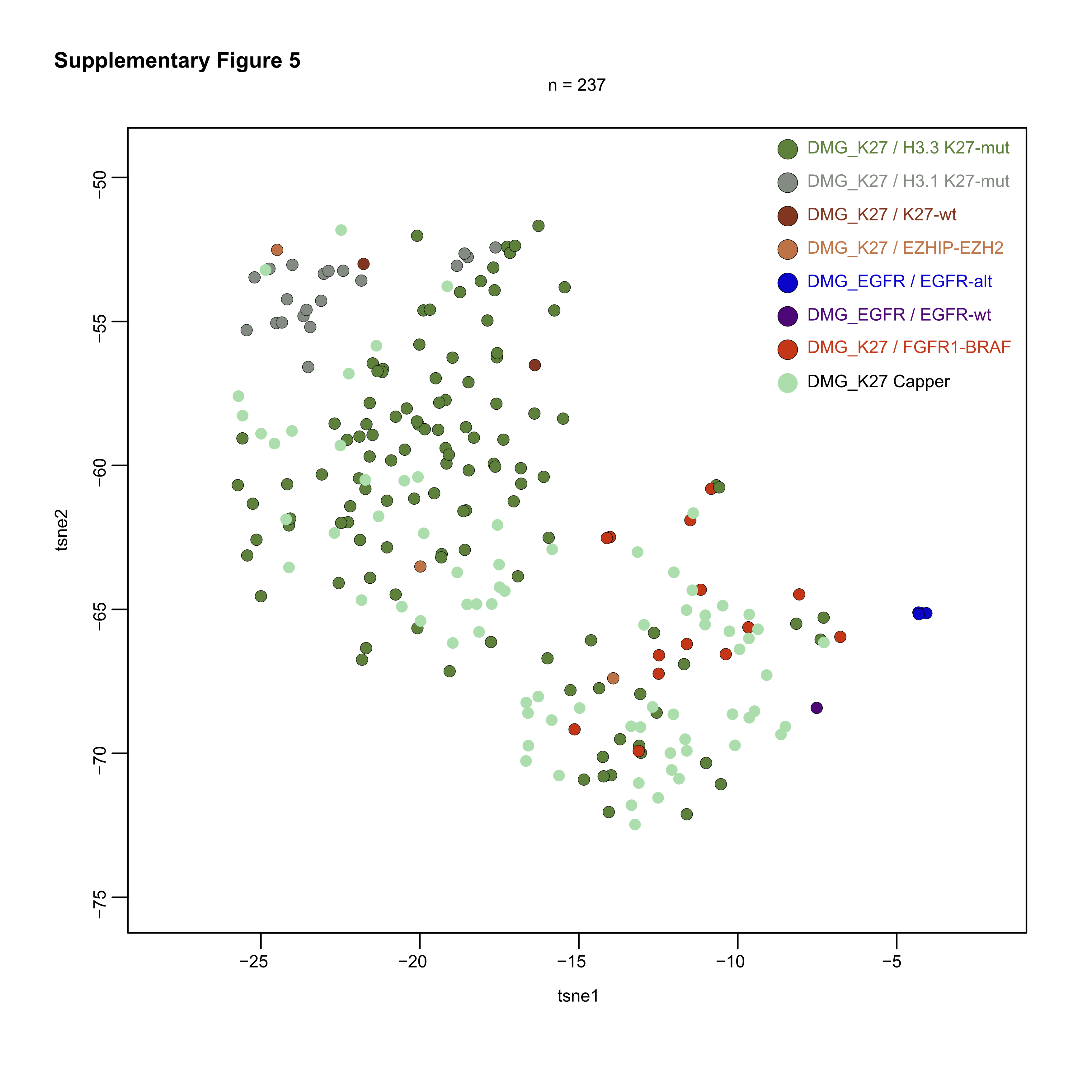

Supplement: Supplementary file 5 — Supplementary file5 Supplementary Fig. 5: Clustering based on whole-genome DNA methylation analysis visualized by t-stochastic neighbor embedding (t-SNE) combined with DMG_K27 samples of the reference cohort from Capper et al. [7]. DMG_K27 Capper = methylation class diffuse midline glioma with Histone 3 K27-alteration included in cohort from Capper; DMG_K27/K27-wt = methylation class diffuse midline glioma Histone 3-altered, without K27M-mutation; DMG_K27/H3.3 K27-mut = methylation class diffuse midline glioma with Histone 3.3 K27M-mutation; DMG_K27/H3.1 K27-mut = methylation class diffuse midline glioma with Histone 3.1 K27M-mutation; DMG_K27/EZHIP-EZH2 = methylation class diffuse midline glioma Histone 3-altered, with EZHIP overexpression or EZH2 alteration; DMG_EGFR/EGFR-alt = methylation class diffuse midline glioma EGFR-altered, with EGFR alteration; DMG_EGFR/EGFR-with = methylation class diffuse midline glioma EGFR-altered, without EGFR alteration; DMG_K27/FGFR1-BRAF = methylation class diffuse midline glioma Histone 3-altered with BRAF V600E or FGFR1 mutation (TIFF 1125 KB) [file 401_2025_2945_MOESM5_ESM.tiff]
