# Supplementary material for: Molecular characterization and clinical features of diffuse midline glioma in the pediatric precision oncology registry INFORM
Source: Acta Neuropathol. 2025 Oct 11;150(1):42. doi: 10.1007/s00401-025-02945-9 (PMC12515216; doi:10.1007/s00401-025-02945-9)
Supplement: Supplementary file 6 — Supplementary file6 Supplementary Table 1: Results of gene set enrichment analysis applying the ‘oncogenic signature gene sets’ of the Human MSigDB Collections. ES = enrichment score; NES = normalized enrichment score; FDR = false discovery rate; FWER = familywise-error rate; Rank at max = Position in ranked list with maximum enrichment score (DOCX 19 KB) [file 401_2025_2945_MOESM6_ESM.docx]

Supplementary Table 1: Results of gene set enrichment analysis applying the ‘oncogenic signature gene sets’ of the Human MSigDB Collections

| **Gene set name** | **Size** | **Enrichment score (ES)** | **Normalized enrichment score (NES)** | **Nominal p-value** | **False discovery rate (FDR) q-value** | **Familywise-error rate (FWER) p-value** | **Rank at Max** | **Leading edge subset defined by** |
| --- | --- | --- | --- | --- | --- | --- | --- | --- |
| KRAS.KIDNEY_UP.V1_UP | 135 | 0.509 | 2.046 | 0.000 | 0.000 | 0.000 | 5507 | tags=36%, list=10%, signal=40% |
| CAHOY_OLIGODENDROCUTIC | 92 | 0.473 | 1.811 | 0.000 | 0.008 | 0.014 | 4684 | tags=34%, list=9%, signal=37% |
| KRAS.PROSTATE_UP.V1_DN | 137 | 0.394 | 1.615 | 0.003 | 0.034 | 0.081 | 5753 | tags=27%, list=11%, signal=30% |
| STK33_SKM_DN | 246 | 0.370 | 1.609 | 0.000 | 0.027 | 0.084 | 5283 | tags=25%, list=10%, signal=28% |
| CTIP_DN.V1_DN | 122 | 0.407 | 1.608 | 0.000 | 0.022 | 0.085 | 10240 | tags=36%, list=19%, signal=44% |
| MEL18_DN.V1_DN | 139 | 0.391 | 1.570 | 0.000 | 0.027 | 0.125 | 6570 | tags=35%, list=12%, signal=40% |
| BMI1_DN.V1_DN | 129 | 0.387 | 1.546 | 0.008 | 0.030 | 0.158 | 6951 | tags=33%, list=13%, signal=37% |
| CSR_LATE_UP.V1_DN | 135 | 0.383 | 1.542 | 0.006 | 0.028 | 0.167 | 5609 | tags=29%, list=10%, signal=32% |
| MYC_UP.V1_DN | 156 | 0.374 | 1.516 | 0.000 | 0.031 | 0.201 | 8360 | tags=36%, list=16%, signal=42% |
| PTEN_DN.V1_UP | 172 | 0.355 | 1.459 | 0.000 | 0.051 | 0.336 | 8412 | tags=29%, list=16%, signal=34% |
| IL21_UP.V1_UP | 174 | 0.356 | 1.458 | 0.000 | 0.047 | 0.336 | 6706 | tags=27%, list=12%, signal=31% |
| KRAS.BREAST_UP.V1_DN | 134 | 0.359 | 1.451 | 0.006 | 0.047 | 0.357 | 6801 | tags=29%, list=13%, signal=33% |
| NOTCH_DN.V1_UP | 175 | 0.343 | 1.438 | 0.000 | 0.049 | 0.399 | 4948 | tags=22%, list=9%, signal=24% |
| ATM_DN.V1_DN | 144 | 0.347 | 1.405 | 0.003 | 0.063 | 0.512 | 9367 | tags=37%, list=17%, signal=44% |
| NRL_DN.V1_UP | 132 | 0.342 | 1.379 | 0.008 | 0.076 | 0.601 | 8064 | tags=30%, list=15%, signal=35% |
| ESC_V6.5_UP_EARLY.V1_UP | 158 | 0.340 | 1.376 | 0.015 | 0.074 | 0.611 | 7857 | tags=32%, list=15%, signal=37% |
| KRAS.600_UP.V1_UP | 270 | 0.314 | 1.374 | 0.000 | 0.072 | 0.620 | 6182 | tags=26%, list=11%, signal=29% |
| JNK_DN.V1_UP | 176 | 0.330 | 1.362 | 0.006 | 0.077 | 0.666 | 5841 | tags=26%, list=11%, signal=29% |
| IL2_UP.V1_DN | 175 | 0.332 | 1.355 | 0.010 | 0.079 | 0.688 | 9887 | tags=34%, list=18%, signal=42% |
| KRAS.AMP.LUNG_UP.V1_UP | 132 | 0.335 | 1.354 | 0.020 | 0.076 | 0.692 | 10246 | tags=36%, list=19%, signal=45% |
| ESC_V6.5_UP_LATE.V1_UP | 176 | 0.331 | 1.353 | 0.009 | 0.073 | 0.698 | 6067 | tags=28%, list=11%, signal=32% |
| JAK2_DN.V1_UP | 172 | 0.330 | 1.351 | 0.006 | 0.071 | 0.711 | 7061 | tags=27%, list=13%, signal=31% |
| KRAS.600.LUNG.BREAST_UP.V1_DN | 267 | 0.313 | 1.350 | 0.003 | 0.069 | 0.716 | 7876 | tags=28%, list=15%, signal=32% |
| SINGH_KRAS_DEPENDENCY_SIGNATURE | 18 | 0.495 | 1.335 | 0.130 | 0.077 | 0.772 | 8867 | tags=33%, list=16%, signal=40% |
| ESC_J1_UP_EARLY.V1_UP | 155 | 0.320 | 1.307 | 0.023 | 0.099 | 0.861 | 8401 | tags=32%, list=16%, signal=38% |
| SNF5_DN.V1_DN | 149 | 0.321 | 1.304 | 0.023 | 0.098 | 0.869 | 9059 | tags=35%, list=17%, signal=42% |
| ALK_DN.V1_DN | 133 | 0.315 | 1.283 | 0.046 | 0.118 | 0.924 | 4928 | tags=21%, list=9%, signal=23% |
| SRC_UP.V1_UP | 159 | 0.310 | 1.282 | 0.041 | 0.115 | 0.928 | 6351 | tags=29%, list=12%, signal=33% |
| KRAS.300_UP.V1_UP | 138 | 0.319 | 1.276 | 0.058 | 0.117 | 0.938 | 6365 | tags=25%, list=12%, signal=28% |
| ESC_J1_UP_LATE.V1_UP | 175 | 0.304 | 1.265 | 0.031 | 0.127 | 0.953 | 6570 | tags=26%, list=12%, signal=29% |
| PRC2_EED_DN.V1_UP | 176 | 0.299 | 1.255 | 0.038 | 0.136 | 0.970 | 4284 | tags=21%, list=8%, signal=23% |
| PRC1_BMI_UP.V1_DN | 174 | 0.302 | 1.246 | 0.058 | 0.142 | 0.977 | 5635 | tags=24%, list=10%, signal=27% |
| LEF1_UP.V1_UP | 189 | 0.299 | 1.245 | 0.056 | 0.139 | 0.978 | 6763 | tags=24%, list=13%, signal=27% |
| KRAS.LUNG.BREAST_UP.V1_DN | 135 | 0.309 | 1.242 | 0.052 | 0.140 | 0.982 | 5874 | tags=24%, list=11%, signal=27% |
| PTEN_DN.V1_DN | 170 | 0.301 | 1.238 | 0.071 | 0.141 | 0.984 | 5914 | tags=22%, list=11%, signal=25% |
| PTEN_DN.V2_UP | 133 | 0.305 | 1.230 | 0.077 | 0.149 | 0.986 | 6246 | tags=24%, list=12%, signal=27% |
| IL15_UP.V1_DN | 166 | 0.295 | 1.224 | 0.065 | 0.154 | 0.987 | 4544 | tags=19%, list=8%, signal=20% |
| NOTCH_DN.V1_DN | 174 | 0.294 | 1.219 | 0.069 | 0.157 | 0.987 | 6912 | tags=21%, list=13%, signal=24% |
| P53_DN.V1_DN | 188 | 0.289 | 1.219 | 0.049 | 0.153 | 0.987 | 5878 | tags=23%, list=11%, signal=26% |
| CAMP_UP.V1_DN | 192 | 0.292 | 1.215 | 0.073 | 0.154 | 0.989 | 7225 | tags=27%, list=13%, signal=31% |
| RPS14_DN.V1_UP | 185 | 0.291 | 1.211 | 0.066 | 0.156 | 0.991 | 9939 | tags=35%, list=18%, signal=42% |
| CYCLIN_D1_KE_.V1_DN | 185 | 0.286 | 1.209 | 0.060 | 0.155 | 0.991 | 8064 | tags=26%, list=15%, signal=30% |
| CAHOY_ASTROCYTIC | 98 | 0.316 | 1.209 | 0.084 | 0.152 | 0.991 | 4315 | tags=22%, list=8%, signal=24% |
| LEF1_UP.V1_DN | 176 | 0.296 | 1.208 | 0.065 | 0.150 | 0.991 | 4268 | tags=20%, list=8%, signal=22% |
| STK33_DN | 249 | 0.283 | 1.205 | 0.046 | 0.151 | 0.993 | 5283 | tags=22%, list=10%, signal=24% |
| AKT_UP.V1_DN | 180 | 0.291 | 1.203 | 0.083 | 0.150 | 0.993 | 9488 | tags=33%, list=18%, signal=40% |
| KRAS.AMP.LUNG_UP.V1_DN | 133 | 0.301 | 1.201 | 0.086 | 0.149 | 0.993 | 5946 | tags=24%, list=11%, signal=27% |
| WNT_UP.V1_UP | 173 | 0.290 | 1.195 | 0.087 | 0.154 | 0.997 | 4647 | tags=18%, list=9%, signal=20% |
| ATM_DN.V1_UP | 143 | 0.292 | 1.195 | 0.102 | 0.151 | 0.997 | 9062 | tags=29%, list=17%, signal=34% |
| KRAS.600_UP.V1_DN | 270 | 0.275 | 1.189 | 0.047 | 0.156 | 0.998 | 5946 | tags=23%, list=11%, signal=25% |
| JAK2_DN.V1_DN | 133 | 0.296 | 1.189 | 0.109 | 0.154 | 0.998 | 6368 | tags=20%, list=12%, signal=22% |
| PGF_UP.V1_DN | 185 | 0.282 | 1.188 | 0.065 | 0.151 | 0.998 | 7292 | tags=23%, list=14%, signal=26% |
| BMI1_DN_MEL18_DN.V1_DN | 139 | 0.298 | 1.187 | 0.096 | 0.151 | 0.998 | 7244 | tags=29%, list=13%, signal=34% |
| STK33_NOMO_DN | 251 | 0.270 | 1.186 | 0.047 | 0.149 | 0.998 | 6483 | tags=24%, list=12%, signal=27% |
| CRX_NRL_DN.V1_UP | 133 | 0.296 | 1.183 | 0.103 | 0.150 | 0.998 | 8064 | tags=29%, list=15%, signal=34% |
| TGFB_UP.V1_UP | 181 | 0.279 | 1.166 | 0.084 | 0.173 | 0.998 | 7799 | tags=27%, list=15%, signal=31% |
| IL21_UP.V1_DN | 176 | 0.281 | 1.162 | 0.104 | 0.175 | 0.998 | 5608 | tags=24%, list=10%, signal=27% |
| PRC2_SUZ12_UP.V1_UP | 178 | 0.277 | 1.159 | 0.098 | 0.176 | 0.998 | 4222 | tags=18%, list=8%, signal=19% |
| P53_DN.V2_UP | 143 | 0.286 | 1.153 | 0.138 | 0.184 | 1.000 | 12169 | tags=41%, list=23%, signal=52% |
| DCA_UP.V1_UP | 175 | 0.277 | 1.151 | 0.137 | 0.185 | 1.000 | 6050 | tags=21%, list=11%, signal=24% |
| VEGF_A_UP.V1_UP | 189 | 0.273 | 1.140 | 0.150 | 0.199 | 1.000 | 7183 | tags=26%, list=13%, signal=30% |
| KRAS.LUNG_UP.V1_DN | 137 | 0.281 | 1.126 | 0.199 | 0.221 | 1.000 | 9334 | tags=28%, list=17%, signal=33% |
| YAP1_UP | 39 | 0.352 | 1.120 | 0.246 | 0.228 | 1.000 | 1339 | tags=15%, list=2%, signal=16% |
| ERBB2_UP.V1_UP | 177 | 0.268 | 1.112 | 0.191 | 0.238 | 1.000 | 5705 | tags=23%, list=11%, signal=25% |
| RELA_DN.V1_DN | 130 | 0.274 | 1.102 | 0.223 | 0.255 | 1.000 | 7861 | tags=25%, list=15%, signal=30% |
| CTIP_DN.V1_UP | 126 | 0.279 | 1.101 | 0.232 | 0.253 | 1.000 | 6205 | tags=22%, list=12%, signal=25% |
| CRX_DN.V1_UP | 125 | 0.275 | 1.092 | 0.218 | 0.266 | 1.000 | 9710 | tags=32%, list=18%, signal=39% |
| PRC1_BMI_UP.V1_UP | 175 | 0.262 | 1.081 | 0.265 | 0.286 | 1.000 | 6923 | tags=23%, list=13%, signal=26% |
| LTE2_UP.V1_UP | 179 | 0.261 | 1.077 | 0.215 | 0.290 | 1.000 | 5953 | tags=21%, list=11%, signal=24% |
| BRCA1_DN.V1_UP | 125 | 0.267 | 1.073 | 0.282 | 0.297 | 1.000 | 7261 | tags=22%, list=14%, signal=25% |
| KRAS.300_UP.V1_DN | 136 | 0.262 | 1.043 | 0.331 | 0.366 | 1.000 | 5946 | tags=23%, list=11%, signal=26% |
| PKCA_DN.V1_DN | 149 | 0.252 | 1.036 | 0.337 | 0.378 | 1.000 | 4787 | tags=18%, list=9%, signal=20% |
| KRAS.BREAST_UP.V1_UP | 132 | 0.260 | 1.033 | 0.364 | 0.381 | 1.000 | 11187 | tags=33%, list=21%, signal=42% |
| PKCA_DN.V1_UP | 153 | 0.254 | 1.030 | 0.354 | 0.383 | 1.000 | 8958 | tags=29%, list=17%, signal=34% |
| KRAS.PROSTATE_UP.V1_UP | 132 | 0.248 | 0.983 | 0.500 | 0.515 | 1.000 | 5245 | tags=17%, list=10%, signal=19% |
| KRAS.KIDNEY_UP.V1_DN | 126 | 0.243 | 0.968 | 0.540 | 0.556 | 1.000 | 7712 | tags=24%, list=14%, signal=28% |
| RELA_DN.V1_UP | 146 | 0.220 | 0.890 | 0.798 | 0.791 | 1.000 | 9690 | tags=29%, list=18%, signal=35% |
| KRAS.50_UP.V1_UP | 47 | 0.260 | 0.883 | 0.666 | 0.799 | 1.000 | 4720 | tags=19%, list=9%, signal=21% |
