# Supplementary material for: Molecular characterization and clinical features of diffuse midline glioma in the pediatric precision oncology registry INFORM
Source: Acta Neuropathol. 2025 Oct 11;150(1):42. doi: 10.1007/s00401-025-02945-9 (PMC12515216; doi:10.1007/s00401-025-02945-9)
Supplement: Supplementary file 7 — Supplementary file7 Supplementary Table 2: List of all applied targeted therapeutic agents (DOCX 12 KB) [file 401_2025_2945_MOESM7_ESM.docx]

Supplementary Table 2: List of all applied targeted therapeutic agents

| Avapritinib |
| --- |
| Bevacizumab |
| Capmatinib |
| Cetuximab |
| Copanlisib |
| Crizotinib |
| Dabrafenib |
| Dasatinib |
| Erdafitinib |
| Everolimus |
| H3K27M vaccine |
| Immunotherapy (not specified) |
| Ipilimumab |
| Larotrectinib |
| Nimotuzumab |
| Nivolumab |
| ONC201 |
| ONC206 |
| Osimertinib |
| Paxalisib |
| Pazopanib |
| Ponatinib |
| Regorafenib |
| Ribociclib |
| Sirolimus |
| Sorafenib |
| Trametinib |
| Vandetanib |
| Vemurafenib |
