# Supplementary material for: Molecular characterization and clinical features of diffuse midline glioma in the pediatric precision oncology registry INFORM
Source: Acta Neuropathol. 2025 Oct 11;150(1):42. doi: 10.1007/s00401-025-02945-9 (PMC12515216; doi:10.1007/s00401-025-02945-9)
Supplement: Supplementary file 8 — Supplementary file8 Supplementary Table 3: Results of univariate analysis. Endpoint = overall survival. OS = overall survival; DMG_K27 = methylation class diffuse midline glioma Histone 3 K27-altered; DMG_EGFR = methylation class diffuse midline glioma EGFR-altered; H3.3 K27 = Histone 3.3 K27M-mutation positive; H3.1 K27 = Histone 3.1 K27M-mutation positive; H3 wt = Histone 3 wildtype; TT = targeted therapy; TKI = tyrosine kinase inhibitor; SOC = standard of care treatment with radiotherapy + temozolomide ± valproic acid (DOCX 15 KB) [file 401_2025_2945_MOESM8_ESM.docx]

Supplementary Table 3: Univariate analysis

| **Variable** | **harzard ratio** | **95% confidence interval** | **overall logrank p-value** |
| --- | --- | --- | --- |
| **localization (n=143) (OS from primary diagnosis)** |  |  |  |
| pons/brainstem (ref) |  |  | 0.04 |
| thalamus/basal ganglia | 0.55 | 0.31-0.99 |  |
| spinal | 0.40 | 0.18-0.90 |  |
| missing data/unclear | 0.74 | 0.45-1.20 |  |
| **methylation group (n=141) (OS from primary diagnosis)** |  |  |  |
| DMG_K27 (ref) |  |  | 0.6 |
| DMG_EGFR | 0.77 | 0.28-2.10 |  |
| **Histone 3 mutation status (n=143)  (OS from primary diagnosis)** |  |  |  |
| H3.3 K27M (ref) |  |  | 0.6 |
| H3.1 K27M | 0.79 | 0.46-1.34 |  |
| H3 wt | 0.85 | 0.39-1.84 |  |
| **TP53 pathway alteration (n=143)  (OS from primary diagnosis)** |  |  |  |
| TP53 wt (ref) |  |  | 0.0003 |
| TP53 altered | 2.01 | 1.36-2.96 |  |
| **MAPK pathway alteration (n=143)  (OS from primary diagnosis)** |  |  |  |
| MAPK pathway not altered (ref) |  |  | 0.03 |
| MAPK pathway altered | 0.57 | 0.34-0.95 |  |
| **BRAF V600E or FGFR1 mutation in K27M-mutated DMG (n=134) (OS from primary diagnosis)** |  |  |  |
| no BRAF V600E or FGFR1 mutation (ref) |  |  | 0.007 |
| BRAF V600E mutation | 0.27 | 0.09-0.76 |  |
| FGFR1 mutation | 0.29 | 0.08-0.99 |  |
| **episode (n=143) (OS from current episode)** |  |  |  |
| primary diagnosis (ref) |  |  | 0.4 |
| 1. relapse | 1.31 | 0.61-2.84 |  |
| ≥ 2. relapse | 1.51 | 0.63-3.62 |  |
| refractory/progressive disease | 1.56 | 0.85-2.85 |  |
| **treatment with TT (n=137) (OS from current episode)** |  |  |  |
| no treatment with TT (ref) |  |  | 0.08 |
| treatment with any TT | 0.72 | 0.50-1.04 |  |
| **treatment with matching TT (n=137) (OS from current episode)** |  |  |  |
| no treatment with TT or treatment with not matching TT (ref) |  |  | 0.02 |
| treatment with matching TT | 0.63 | 0.42-0.94 |  |
| **treatment with TT based on target (n=137)  (OS from current episode)** |  |  |  |
| no treatment with TT (ref) |  |  | 0.2 |
| treatment with TT matching to high priority target | 0.63 | 0.36-1.10 |  |
| treatment with TT matching to low priority target | 0.62 | 0.36-1.06 |  |
| treatment with TT not matching to target | 0.97 | 0.59-1.59 |  |
| **treatment with ONC201 (n=137)  (OS from current episode)** |  |  |  |
| no treatment with ONC201 (ref) |  |  | 0.09 |
| treatment with ONC201 | 0.63 | 0.37-1.09 |  |
| **treatment with TKI based on target (n=137)  (OS from current episode)** |  |  |  |
| no TT (ref) |  |  | 0.06 |
| TT matching to target | 0.63 | 0.41-0.97 |  |
| TT not matching | 1.13 | 0.68-1.88 |  |
| **treatment with TT for MAPK-altered cases (n=21)  (OS from current episode)** |  |  |  |
| MAPK altered - no TT (ref) |  |  | 0.5 |
| MAPK altered - matching TT | 0.65 | 0.21-2.04 |  |
| MAPK altered - not matching TT | 1.31 | 0.26-4.80 |  |
| **treatment with SOC versus matching TT (n=81)**  **(OS from primary diagnosis)** |  |  |  |
| treatment with SOC (ref) |  |  | 0.03 |
| treatment with matching TT | 0.59 | 0.36-0.96 |  |
